# Single-nucleus transcriptomic landscape of primate hippocampal aging

**DOI:** 10.1007/s13238-021-00852-9

**Published:** 2021-05-30

**Authors:** Hui Zhang, Jiaming Li, Jie Ren, Shuhui Sun, Shuai Ma, Weiqi Zhang, Yang Yu, Yusheng Cai, Kaowen Yan, Wei Li, Baoyang Hu, Piu Chan, Guo-Guang Zhao, Juan Carlos Izpisua Belmonte, Qi Zhou, Jing Qu, Si Wang, Guang-Hui Liu

**Affiliations:** 1 State Key Laboratory of Membrane Biology, Institute of Zoology, Chinese Academy of Sciences, Beijing, 100101 China; 2 State Key Laboratory of Stem Cell and Reproductive Biology, Institute of Zoology, Chinese Academy of Sciences, Beijing, 100101 China; 3 Advanced Innovation Center for Human Brain Protection, National Clinical Research Center for Geriatric Disorders, Xuanwu Hospital Capital Medical University, Beijing, 100053 China; 4 CAS Key Laboratory of Genomic and Precision Medicine, Beijing Institute of Genomics, Chinese Academy of Sciences, Beijing, 100101 China; 5 Institute for Stem Cell and Regeneration, Chinese Academy of Sciences, Beijing, 100101 China; 6 University of Chinese Academy of Sciences, Beijing, 100049 China; 7 China National Center for Bioinformation, Beijing, 100101 China; 8 Aging Translational Medicine Center, Xuanwu Hospital, Capital Medical University, Beijing, 100053 China; 9 Department of Neurosurgery, Xuanwu Hospital, Capital Medical University, Beijing, 100053 China; 10 Sino-Danish College, University of Chinese Academy of Sciences, Beijing, 101408 China; 11 Sino-Danish Center for Education and Research, Beijing, 101408 China; 12 Department of Obstetrics and Gynecology, Center for Reproductive Medicine, Peking University Third Hospital, Beijing, 100191 China; 13 Beijing Institute for Stem Cell and Regenerative Medicine, Beijing, 100101 China; 14 Stem Cell Research Center, Peking University Third Hospital, Beijing, 100191 China; 15 Gene Expression Laboratory, Salk Institute for Biological Studies, La Jolla, CA USA

**Keywords:** aging, hippocampus, primate, single-cell RNA sequencing

## Abstract

The hippocampus plays a crucial role in learning and memory, and its progressive deterioration with age is functionally linked to a variety of human neurodegenerative diseases. Yet a systematic profiling of the aging effects on various hippocampal cell types in primates is still missing. Here, we reported a variety of new aging-associated phenotypic changes of the primate hippocampus. These include, in particular, increased DNA damage and heterochromatin erosion with time, alongside loss of proteostasis and elevated inflammation. To understand their cellular and molecular causes, we established the first single-nucleus transcriptomic atlas of primate hippocampal aging. Among the 12 identified cell types, neural transiently amplifying progenitor cell (TAPC) and microglia were most affected by aging. In-depth dissection of gene-expression dynamics revealed impaired TAPC division and compromised neuronal function along the neurogenesis trajectory; additionally elevated pro-inflammatory responses in the aged microglia and oligodendrocyte, as well as dysregulated coagulation pathways in the aged endothelial cells may contribute to a hostile microenvironment for neurogenesis. This rich resource for understanding primate hippocampal aging may provide potential diagnostic biomarkers and therapeutic interventions against age-related neurodegenerative diseases.

**Supplementary Information:**

The online version contains supplementary material available at 10.1007/s13238-021-00852-9.

## Introduction

Aging is a major risk factor for human neurodegenerative disorders, such as Alzheimer’s disease (AD), which is rapidly rising in prevalence with age and has become the most pressing challenge to be tackled. The hippocampus plays a crucial role in spatial and episodic learning and memory (Morrison and Baxter, 2012; Fan et al., 2017; Kuhn et al., 2018). Age-related decline in hippocampal functions manifests as a profound yet inevitable impairment in cognitive abilities and increases vulnerability to AD (Morrison and Baxter, 2012; Fan et al., 2017; Hou et al., 2019). Therefore, a comprehensive understanding of the underlying mechanisms of hippocampal aging is of scientific and clinical importance.

Although still being debated, adult neurogenesis continues in the subgranular zone (SGZ) of the dentate gyrus (DG) within the mammalian hippocampus (Boldrini et al., 2018), critical to contextual learning and episodic memory (Kuhn et al., 2018; Navarro Negredo et al., 2020). In the adult DG, radial glia-like quiescent neural stem cell (NSC) allows for the addition of newborn neuron throughout life, initiating the neurogenesis trajectory through the transition into activation, proliferation, and then generation of transiently amplifying progenitor cell (TAPC) (Kempermann et al., 2015; Li et al., 2015; Navarro Negredo et al., 2020). TAPC then gives rise to neuroblast, which further differentiates into immature and then mature granule neuron or glial cell (Aimone et al., 2014; Encinas et al., 2011; Navarro Negredo et al., 2020). Both NSC and their progenies interact extensively with several kinds of neurogenic niche cells, including oligodendrocyte, microglia, and cells comprising the neurovasculature (Aimone et al., 2014; Fan et al., 2017). Although a decline in the volume of the aged anterior hippocampus was observed in some studies (Malykhin et al., 2008), others reported conflicting findings or altered hippocampal shape rather than its volume during the aging process (Head et al., 2005; Yang et al., 2013). Through histological analysis, compromised neurogenesis, a gradual loss of synaptic plasticity, elevated microglia activation, and diminished angiogenesis have been identified as major age-associated changes in the hippocampus, which may result in age-dependent cognitive decline (Leuner et al., 2007; Morrison and Baxter, 2012; Fan et al., 2017; Navarro Negredo et al., 2020). Although extensive studies have been reported on the morphological characteristics of the hippocampus during aging, the complexity of underlying cellular and molecular alterations has never been revealed comprehensively and at single-cell resolution.

Due to ethical restrictions, it is difficult to obtain disease-free human brain tissues, including the hippocampus from both young and old individuals. Therefore, unbiased studies of human hippocampal degeneration are not feasible. Since non-human primates (NHPs) share similar genetic, physiological, and neurological characteristics with humans (Chen et al., 2012, 2016; Chen et al., 2017; Colman, 2018; Zhang et al., 2018), especially similar neurogenesis within the SGZ of the DG (Leuner et al., 2007), they represent ideal models for the study of primate hippocampal aging. On the other hand, the high complexity in its cellular composition requires the dissection of molecular mechanisms underlying hippocampal aging and cognitive decline to be performed at single-cell resolution (He et al., 2020; Zhong et al., 2020). Emerging single-cell/nucleus RNA sequencing (scRNA-seq/snRNA-seq) techniques have unraveled the transcriptional alterations underlying the heterogeneous process of aging at cell-type-specific resolution in multiple organs (He et al., 2020; Li et al., 2020; Ma et al., 2020a, b; Wang et al., 2020a, b). Of note, snRNA-seq provides unique advantages in analyzing tissues difficult to be dissociated, such as the neuron-rich hippocampus, thus reducing the bias in cellular capture and the accompanied transcriptional artifacts (Habib et al., 2016; He et al., 2020; Ma et al., 2020a). However, snRNA-seq has not been applied to explore the cellular and molecular alterations of hippocampal aging in the primates.

In this study, we demonstrated an array of aging-associated damages in the NHP hippocampus, including genomic and epigenomic instability, loss of proteostasis, as well as increased inflammation. To explore unique cellular and molecular characteristics underlying these age-related phenotypes, we generated a high-resolution single-nucleus transcriptomic atlas of hippocampal aging in NHPs. This atlas enabled us to identify the transcriptional alterations underlying the early onset of dysregulation in adult hippocampal neurogenesis, and to unveil the contributing factors to a hostile microenvironment for neurogenesis in the aged hippocampus. Overall, our study deposited a valuable resource for the identification of new diagnostic biomarkers and potential therapeutic targets for interventions against hippocampal aging and related human neurodegenerative disorders.

## Results

### Accumulation of aging-associated damages in the aged hippocampus from cynomolgus monkeys

**Figure 1:**
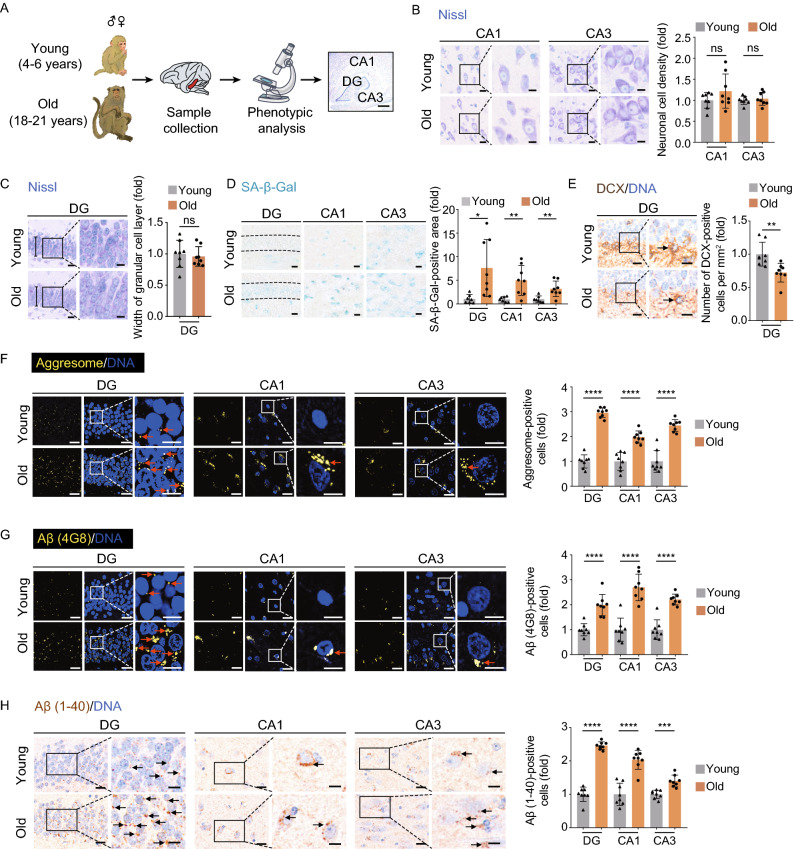
**Aging-related phenotypes of the cynomolgus monkey hippocampus**. (A) Flow chart of the phenotypic analysis on the hippocampal tissues collected form young and old monkeys. Representative image of Nissl staining in the hippocampus is shown (right panel, dentate gyrus (DG), CA1 region (CA1) and CA3 region (CA3), scale bar, 700 μm). (B) Nissl staining in CA1 and CA3 regions of the hippocampus from young and old monkeys. Representative images are shown on the left; neuronal cell densities in corresponding regions are quantified as fold changes (old vs. young), shown as means ± SEM on the right. Scale bars, 20 μm and 10 μm (zoomed-in image). Young, *n* = 8; old, *n* = 8 monkeys. ns, not significant. (C) Nissl staining in the dentate gyrus (DG) from young and old monkeys. Representative images are shown on the left; the widths of the granular cell layers in the DG are quantified as fold changes (old vs. young), shown as means ± SEM on the right. Scale bars, 20 μm and 10 μm (zoomed-in image). Young, *n* = 8; old, *n* = 8 monkeys. ns, not significant. (D) SA-β-Gal staining in the indicated regions of the hippocampus from young and old monkeys. Representative images are shown on the left; SA-β-Gal positive areas in the DG, CA1 and CA3 regions are quantified as fold changes (young vs. old), shown as means ± SEM on the right. Scale bar, 20 μm. Young, *n* = 7; old, *n* = 8 monkeys. *P < 0.05; **P < 0.01. (E) Immunohistochemical staining of DCX in the DG region of the hippocampus from young and old monkeys. Representative images are shown on the left; DCX-positive cells are quantified as fold changes of their numbers in the old DG vs. in young counterparts, shown as means ± SEM on the right. Black arrows indicate the DCX-positive cells. Scale bars, 20 μm and 10 μm (zoomed-in image). Young, *n* = 8; old, *n* = 8 monkeys. **P < 0.01. (F) Aggresome staining in the indicated regions of the hippocampus from young and old monkeys. Representative images are shown on the left; aggresome-positive cells are quantified as fold changes of their numbers in the old DG, CA1 and CA3 regions vs. in young counterparts, shown as means ± SEM on the right. Red arrows indicate the aggresome-positive cells. Scale bars, 20 μm and 10 μm (zoomed-in image). Young, *n* = 8; old, *n* = 8 monkeys. ****P < 0.0001. (G) Immunofluorescence staining of Aβ (4G8) accumulation in the indicated regions of the hippocampus from young and old monkeys. Representative images are shown on the left; Aβ (4G8)-positive cells are quantified as fold changes of their numbers in the old DG, CA1 and CA3 regions vs. young counterparts, shown as means ± SEM on the right. Red arrows indicate the Aβ (4G8)-positive cells. Scale bars, 20 μm and 10 μm (zoomed-in image). Young, *n* = 8; old, *n* = 8 monkeys. ****P < 0.0001. (H) Immunofluorescence staining of Aβ (1-40) accumulation in the indicated regions of the hippocampus from young and old monkeys. Representative images are shown on the left; quantitative data for the relative Aβ (1-40)-positive cells in the DG, CA1 and CA3 regions are shown as means ± SEM on the right. The relative fold of number of Aβ (1-40)-positive cells was obtained by normalizing the number of Aβ (1-40)-positive cells of the old monkey with the young monkey. Black arrows indicate the Aβ (1-40)-positive cell. Scale bars, 20 μm and 10 μm (zoomed-in image). Young, *n* = 8; old, *n* = 8 monkeys. ***P < 0.001, ****P < 0.0001

**Figure 2:**
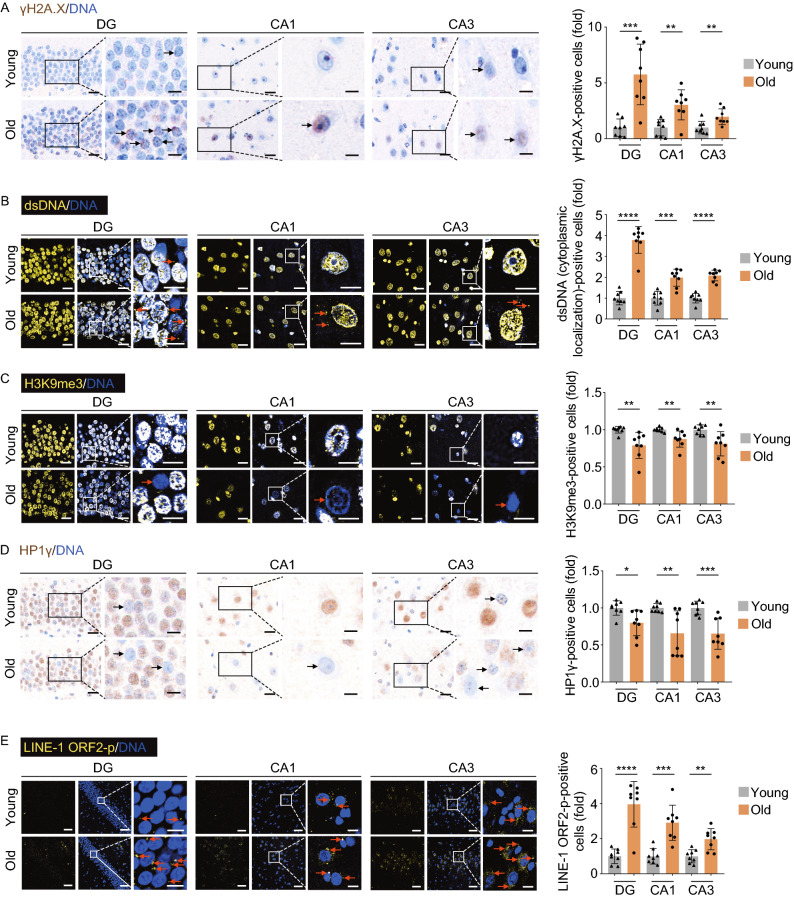
**Aging-related loss of genomic and epigenomic stability in the monkey hippocampus**. (A) Immunohistochemical staining of γH2A.X in the indicated regions of the hippocampus from young and old monkeys. Representative images are shown on the left; γH2A.X-positive cells are quantified as fold changes of their numbers in the old DG, CA1 and CA3 regions vs. in young counterparts, shown as means ± SEM on the right. Black arrows indicate the γH2A.X-positive cells. Scale bars, 20 μm and 10 μm (zoomed-in image). Young, *n* = 8; old, *n* = 8 monkeys. ***P < 0.001; **P < 0.01. (B) Immunofluorescence staining of dsDNA in the indicated regions of the hippocampus from young and old monkeys. Representative images are shown on the left; cytoplasmic dsDNA-positive cells are quantified as fold changes of their numbers in the old DG, CA1 and CA3 regions vs. in young counterparts, shown as means ± SEM on the right. Red arrows indicate the cytoplasm-localized dsDNA. Scale bars, 20 μm and 10 μm (zoomed-in image). Young, *n* = 8; old, *n* = 8 monkeys. ****P < 0.0001; ***P < 0.001. (C) Immunofluorescence staining of H3K9me3 in the indicated regions of the hippocampus from young and old monkeys. Representative images are shown on the left; H3K9me3-positive cells are quantified as fold changes of their numbers in the old DG, CA1 and CA3 regions vs. in young counterparts, shown as means ± SEM on the right. Red arrows indicate the H3K9me3-negative cells. Scale bars, 20 μm and 10 μm (zoomed-in image). Young, *n* = 8; old, *n* = 8 monkeys. **P < 0.01. (D) Immunofluorescence staining of HP1γ in the indicated regions of the hippocampus from young and old monkeys. Representative images are shown on the left, arrows indicate HP1γ -negative cells; while HP1γ-positive cells in the old DG, CA1 and CA3 regions vs. young regions are quantified as fold changes (means ± SEM) on the right. Black arrows indicate the HP1γ-negative cells. Scale bars, 20 μm and 10 μm (zoomed-in image). Young, *n* = 8; old, *n* = 8 monkeys. **P < 0.01. (E) Immunofluorescence staining of LINE-1 ORF2p in the indicated regions of the hippocampus from young and old monkeys. Representative images are shown on the left; LINE-1 ORF2p-positive cells are quantified as fold changes of their numbers in the old DG, CA1 and CA3 regions vs. in young counterparts, shown as means ± SEM on the right. Red arrows indicate the LINE-1 ORF2p-positive cells. Scale bars, 50 μm and 10 μm (Zoomed in image). Young, *n* = 8; old, *n* = 8 monkeys. ****P < 0.0001

To unravel the structural and functional alterations during physiological aging, we first isolated hippocampal tissues from eight young (4–6 years old) and eight aged (18–21 years old) cynomolgus monkeys, which are approximately ~16 and ~60 years old in human age, respectively (Figs. 1A and S1A) (Li et al., 2020; Ma et al., 2020b; Wang et al., 2020a, b; Zhang et al., 2020c). We compared different anatomical regions of the hippocampus, including CA1, CA3, and the dentate gyrus (DG), and found their width and overall cell density were comparable to those in the young counterparts (Figs. 1B, 1C and S1B). However, all three regions showed an increase in cellular senescence, as the proportions of senescence-associated β-galactosidase (SA-β-Gal) staining-positive cells escalated with age (Fig. 1D). In line with the notion that neurogenesis decay was usually defined as one of the hippocampal aging hallmarks (Navarro Negredo et al., 2020), we observed a decrease of DCX-positive neural progenitor cells/neuroblasts in the subgranular zone (SGZ) of the hippocampal DG (Fig. 1E) (Artegiani et al., 2017; Mauffrey et al., 2019). Moreover, we found aging-associated accumulation of a broad range of protein aggregates and amyloid-β (Aβ) deposits (immunostained by the pan-specific anti-Aβ antibody (4G8) and the anti-Aβ40 antibody) (Fig. 1F–H) (Brunk and Terman, 2002; Buckig et al., 2002; Li et al., 2004; Wegiel et al., 2012; Giacobini and Gold, 2013). Importantly, we also found an accompanying increase in genomic and epigenomic instability in these regions, such as increased DNA damage (marked by γ-H2A.X foci formation), increased cytosolic dsDNA release from the nucleus, loss of heterochromatin (marked by H3K9me3 and HP1γ reduction) and retrotransposon activation (marked by LINE-1 ORF2p accumulation) (Fig. 2A–E) (De Cecco et al., 2019; Geng et al., 2019; Hu et al., 2020; Ma et al., 2020a; Zhang et al., 2020b). Altogether, these results supported that the NHP hippocampus suffered from extensive aging-associated damages during physiological aging even without obvious overall structural disorganization.

### Construction of a single-nucleus transcriptomic atlas of the NHP hippocampus

**Figure 3:**
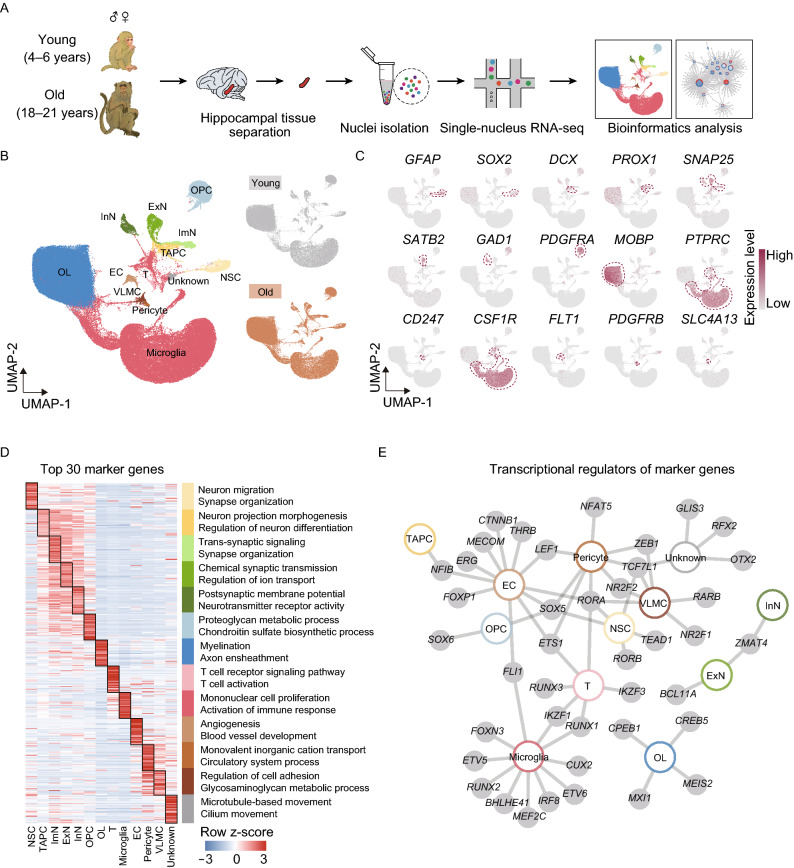
**Construction of single-nucleus transcriptomic atlas of the monkey hippocampus**. (A) Flow chart of snRNA-seq and bioinformatics analysis of the monkey hippocampus. Young, *n* = 7; old, *n* = 8 monkeys. (B) Left, UMAP plot showing distribution of different cell types in the monkey hippocampus. Right, UMAP plots showing distribution of different cell types in the young (top) and old (bottom) hippocampus. (C) UMAP plots showing the expression profiles of indicated cell-type-specific marker genes of corresponding cell types in the monkey hippocampus. (D) Heatmap showing the expression profiles of top 30 cell-type-specific marker genes of different cell types in the monkey hippocampus with their enriched functional annotations on the right. (E) Network plot showing transcriptional regulators of cell-type-specific marker genes (adjusted *P*-value < 0.05, |logFC| > 1) of different cell types in the monkey hippocampus

To investigate the cellular and molecular signatures underlying hippocampal aging, we applied the snRNA-seq technique to capture all cell types in the hippocampus. After stringent filtration, 116,951 qualified single-nucleus transcriptomes were retained with a median of 1,972 genes per nucleus from young and old individuals for downstream analysis (Figs. 3A, 3B and S1C). No apparent difference in cell type distribution was observed among age and sex groups (Fig. S1D), guaranteeing the reliability of gene expression profiling. Uniform Manifold and Projection (UMAP) algorithm analysis identified a total of 12 cell types based on their unique gene-expression signatures (Fig. 3B, 3C and Table S1). Consistent with previous reports (Boldrini et al., 2018), the hippocampal cell populations were constituted by three major cell types, including neurogenic lineage cells, oligodendrocyte lineage cells and niche cells. Neurogenic lineage cells include neural stem cells (NSC) (*GFAP*^*+*^ and *SOX2*^*+*^), transiently amplifying progenitor cells (TAPC) (*DCX*^*+*^, *PROX1*^*+*^ and *SNAP25*^*-*^), immature neurons (ImN) (*PROX1*^*+*^ and *SNAP25*^*+*^), excitatory neurons (ExN) (*SATB2*^*+*^ and *SNAP25*^*+*^), and inhibitory neurons (InN) (*GAD1*^*+*^ and *SNAP25*^*+*^) (Fig. 3B and 3C). Oligodendrocyte lineage cells comprise two cell types, the oligodendrocyte progenitor cells (OPC) (*PDGFRA*^*+*^), and oligodendrocytes (OL) (*MOBP*^*+*^) (Fig. 3B and 3C). As for niche cells, they were composed of T cells (T) (*CD247*^*+*^), microglia (*CSF1R*^*+*^), endothelial cells (EC) (*FLT1*^*+*^), pericytes (*PDGFRB*^*+*^), vascular leptomeningeal cells (VLMC) (*SLC4A13*^*+*^) (Fig. 3B and 3C). In addition to canonical markers, we also identified a set of novel markers for each cell type (Table S2). Gene Ontology (GO) analysis of these cell-type-specific marker genes demonstrated functional characteristics of the corresponding cell type in the hippocampus (Fig. 3D and Table S1). For example, “Neuron migration” was enriched for the top 30 marker genes for NSC, “Regulation of neuron differentiation” for TAPC, “Synapse organization” for ImN, “Chemical synaptic transmission” for ExN, “Postsynaptic membrane potential” for InN, “Proteoglycan metabolic process” for OPC, and “Myelination” for OL, etc.

Moreover, we constructed a regulatory network of transcription factors (TFs), which defined core TFs unique for each cell type and hub TFs shared by at least two cell types (Fig. 3E and Table S1). For instance, the prominent genes comprising the cell-type-specific TF network included *TEAD1* for NSC, *SOX6* for OPC, *CREB5* for OL, and so on (Fig. 3E). Meanwhile, *ZMAT4* was shared by ExN and InN, suggesting its essential roles in neuron specification and functional maintenance. *SOX5* was shared by NSC, OPC, EC and pericyte, indicative of its function as a broad-acting transcriptional regulator. The network depicted the unique and coordinated transcriptional regulations for the establishment of hippocampal cell identities. Taken together, this atlas allows for, in our following analysis, a comprehensive delineation of the cellular and molecular alterations induced by aging in the NHP hippocampus.

### Characterization of the aging-associated cellular and molecular profiles of the NHP hippocampus

**Figure 4:**
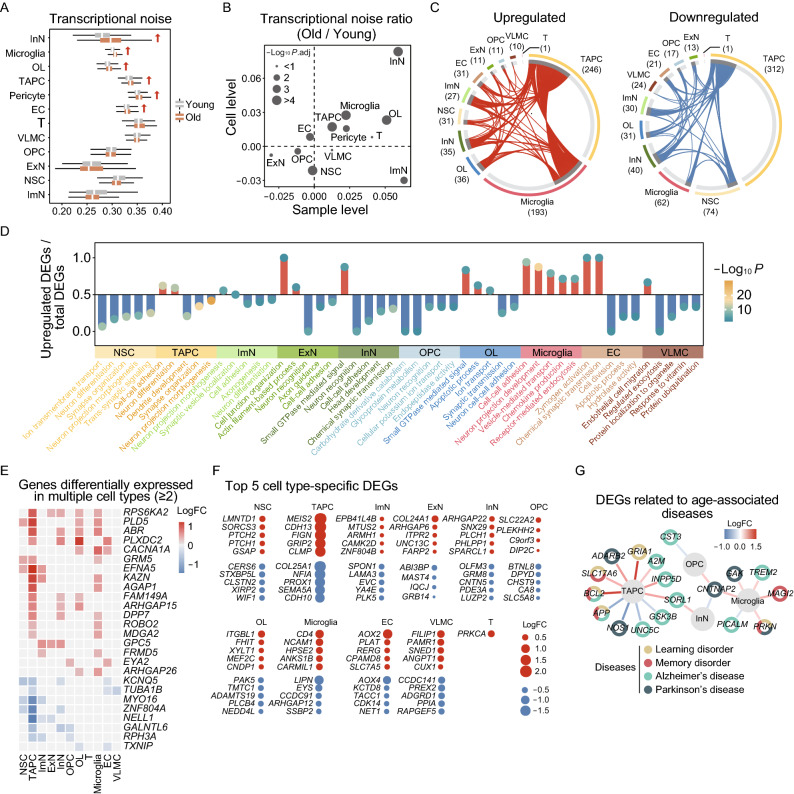
**Cellular and molecular aging characteristics of the aged monkey hippocampus**. (A) Boxplot showing transcriptional noise in different cell types in young and old monkey hippocampus. Arrows indicate cell types whose transcriptional noise is significantly increased in the aged groups. (B) Scatter plot showing the log_2_ ratio of transcriptional noise of different cell types in the monkey hippocampus between the old and young groups at the cell and sample levels. (C) Circos plots showing aging-related up- and down-regulated differentially expressed genes (DEGs) (adjusted *P*-value < 0.05, |logFC| > 0.25) of different cell types in the monkey hippocampus. Each connecting curve represents a gene that is up- or down-regulated in two cell types. (D) Bar plot showing GO terms enriched for aging-related DEGs of different cell types in the monkey hippocampus. Y axis represents the ratio of upregulated genes to total DEGs in corresponding terms. (E) Heatmap showing genes differentially expressed in at least two cell types in the monkey hippocampus. Only genes with same direction of differential expression among different cell types are included. (F) Dot plots showing top five cell-type-specific DEGs of different cell types. Only those with annotations are showed. Red dots represent upregulated genes and blue dots represent downregulated ones. (G) Network plot showing DEGs associated with aging-related diseases in different cell types in the monkey hippocampus

To dissect cell-type-specific aging-related alterations in gene expression, we first examined gene expression signatures of marker genes for each cell type in the NHP hippocampus. The unchanged expression signatures suggested that cell identities remain relatively stable during aging (Fig. S2A). Yet, given the profound genomic and epigenomic destabilization observed in various hippocampal regions (Fig. 2), we wondered if there are similar transcriptional instabilities with age. As expected, calculation of the transcriptional noise indicated that TAPC and InN in the neurogenic lineage cells, OL in the oligodendrocyte lineage cells, as well as microglia, pericyte and EC in the niche cells exhibited higher transcriptional noise with age (Fig. 4A and 4B), which was in agreement with the increased transcriptional noise observed in other aged tissues (Li et al., 2020; Wang et al., 2020a; Zhang et al., 2020c). In addition, to annotate the hotspot genes involved in aging and age-related diseases, we performed an integrative comparative analysis between cell-type-specific marker genes and genes from the Aging Atlas (AA) gene set and gene sets implicated in learning and memory disorders (LD, MD) as well as neurodegenerative diseases including Alzheimer’s disease (AD) and Parkinson’s disease (PD) (Aging Atlas, 2021). The marker genes of microglia and EC were the most frequently annotated as hotspot genes (Fig. S2B), indicating that these cell types may be more susceptible to aging and age-related diseases.

In order to further identify the critical cell types affected by and/or responsible for hippocampal aging, we analyzed age-associated differentially expressed genes (DEGs) across all cell types. The largest numbers of DEGs were observed in TAPC, microglia, and NSC (246, 193 and 31 upregulated genes, 312, 62 and 74 downregulated genes, respectively in aged tissues) (Fig. 4C and Table S2). In addition, the numbers of DEGs in the male group were larger than their counterparts in the female group across almost all cell types (Fig. S2C), suggesting that the male hippocampus may be more susceptible to aging. Further, through functional annotation enrichment analysis, we investigated molecular pathways most affected by aging in these cell types. We noticed that “neuron differentiation” was enriched for downregulated DEGs in both NSC and ImN, suggesting for the early onset of dysregulation of genes related to neurogenesis. Secondly, increased expression of genes involved in “cell junction organization” and “actin filament-based process” in ExN (Fig. 4D) implied for the correlation between a dysregulation of the cytoskeleton and compromised synaptic dynamics with age (Végh et al., 2014). Also contributing to age-related synaptic degeneration are oligodendrocyte lineage cells, as GO analysis of DEGs in OPC and OL revealed increased “apoptotic process”, along with decreased “neuron recognition” and “synaptic transmission” (Fig. 4D). Lastly, for niche cells, increased “chemokine production” and “receptor-mediated endocytosis” in microglia, together with the elevated “zymogen activation” and “endothelial cell migration” in EC and VLMC (Fig. 4D), pointed to elevated inflammation and debris engulfment along with compromised supporting abilities with age.

In addition, a total of 18 genes were consistently upregulated, along with 8 consistently downregulated ones in at least two cell types (Fig. 4E). For instance, *ROBO2*, which encodes roundabout guidance receptor 2 and promotes pro-inflammatory immune response (Martinelli and Real, 2019), is upregulated in aged TAPC and microglia (Fig. 4E), suggesting elevated inflammation in the aged hippocampus (Fig. 4D). *NELL1*, which encodes a cytoplasmic protein that contains epidermal growth factor (EGF)-like repeats and is involved in cell growth regulation and differentiation (Nakamura et al., 2012), is downregulated in TAPC and derivative neurons (Fig. 4E), correlative with the aberrant neurogenesis. On the other hand, for cell-type-specific DEGs, we listed the top 5 genes for each cell type and noticed that their dysregulation might compromise corresponding cellular functions (Fig. 4F and Table S2). For instance, *GSAP*, an upregulated gene in aged NSC, was reported to be responsible for promoting amyloid-beta (Aβ) production through interaction with both gamma-secretase and its substrate (He et al., 2010), thus probably contributing to the increased Aβ deposits observed in the aged hippocampus (Fig. 1F). *CDH13*, upregulated in aged TAPC, encodes a calcium-dependent cell adhesion protein with a central role in the regulation of brain network development and synaptic plasticity, and hence is associated with neuropsychriatric disorders (Rivero et al., 2013). *GRM8*, downregulated in aged InN, encodes a glutamate metabotropic receptor of neurotransmitter L-glutamate, and is known to facilitate neuronal resilience to central nervous system (CNS) inflammation (Woo et al., 2021). *PAK5* was downregulated in OL, which may impair cytoskeletal remodeling, and compromise the synaptic vesicle trafficking and axon-myelin contacts (Zhang et al., 2020a). These dysregulated genes may underlie the progressive functional decay of hippocampal cells during aging.

Next, we performed an integrative comparative analysis between newly identified age-associated DEGs of the NHP hippocampus and annotated hotspot genes from various human neurodegenerative disease-associated gene sets or from the Aging Atlas gene set (Fig. 4G) (Aging Atlas, 2021). Through the construction of a high-risk DEG network linking hippocampal aging and neurodegenerative diseases, we found that most of the high-risk DEGs were enriched in TAPC and microglia. This network suggested that these two cell types were more susceptible to age-related diseases, especially for AD, as exemplified by *TREM2*, *INPP5D*, *SORL1*, etc. (Fig. 4G). Among them, *TREM2* encodes an essential microglia sensor that mediates their response to environmental signals (Leyns et al., 2017), and its upregulation in microglia is closely related to inflammatory responses and pathogenesis of AD (Keren-Shaul et al., 2017; Ulland and Colonna, 2018). Meanwhile, some high-risk DEGs were associated with multiple disorders (Fig. 4G). For example, *PRKN* is a risk factor for both learning and memory disorders, as well as PD, as it encodes a component of a multiprotein E3 ubiquitin ligase complex (Lubbe et al., 2021), whose upregulation may be linked to the misregulation of proteostasis in the aged hippocampus (Fig. 1F) (Costa-Mattioli and Walter, 2020). Among these high-risk genes, those dysregulated in more than one cell type, such as *CNTNAP2* and *SORL1* (Fig. 4G), may represent potential targets to defer the onset of cognitive decline and neurodegenerative diseases in the elderly.

In addition to intrinsic changes, direct cell-to-cell contacts also modulate neurogenesis during aging (Ransohoff, 2016; Leng and Edison, 2021), which may precede the decline in neurogenesis. Thus, we preformed cell-cell interaction analysis across all cell types and discovered a global increase in cell-cell interactions in the aged hippocampus compared to the young counterparts (Fig. S2E, S2F and Table S3), which was consistent with the observations in other aged tissues (Ma et al., 2020a, b). Of note, VLMC, TAPC, and OPC were the top three cell types that display the most old-specific cell-cell interactions; while TAPC, EC and pericyte were the top three cell types that demonstrate the most young-specific interactions (Fig. S2E and S2F), pinpointing the aberrant communications between the neural progenitor cells and neurogenic niche cells, which may contribute to the compromised neurogenesis.

Altogether, our findings identified aging-related molecular features of the NHP hippocampus, demonstrating impaired neurogenesis and neuronal function, increased inflammatory response as the most affected and potential hallmarks in the age-related neurodegeneration of the hippocampus.

### Profiling of aging-related molecular alterations along the differentiation trajectories in hippocampal neurogenesis

**Figure 5:**
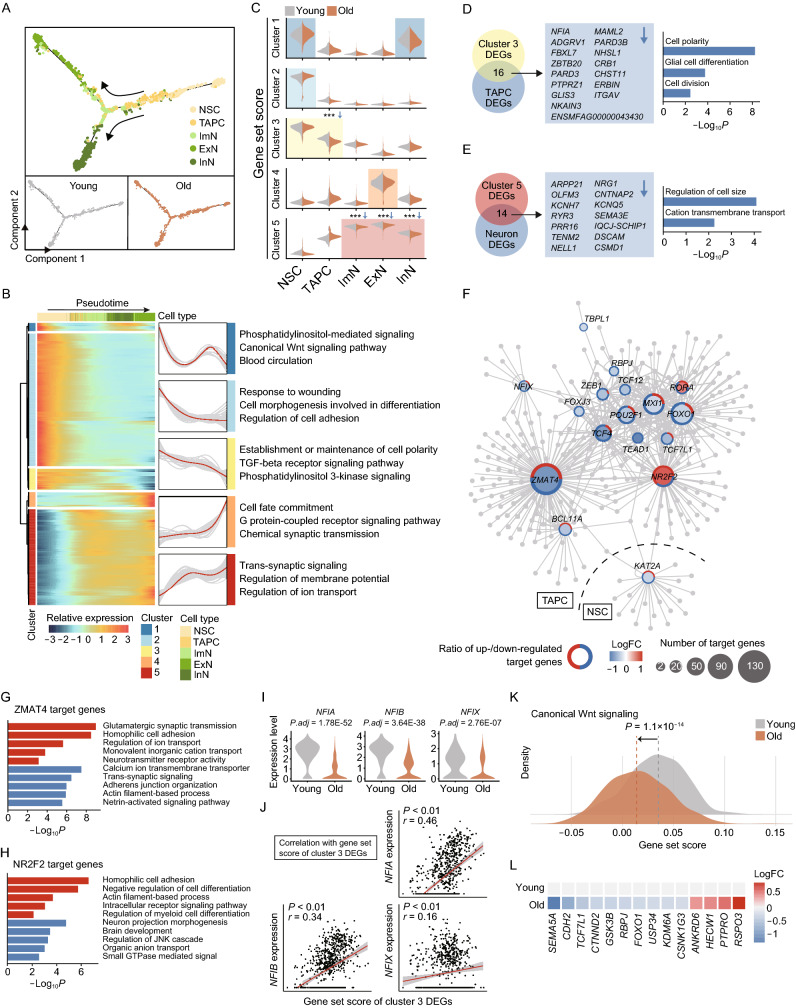
**Aging-related cellular and molecular alterations along the trajectories of the neurogenesis**. (A) Pseudotime analysis of the neurogenic lineage cells in the monkey hippocampus. The points are colored by cell types (top) and age (bottom). The arrows indicate the directions of differentiation trajectories. (B) Heatmap showing the expression profiles along the pseudotime of top 500 DEGs (q value < 1 × 10^−4^), which were divided into five clusters with the expression pattern and enriched GO terms of the corresponding cluster represented on the right. (C) Violin plots showing gene set scores of indicated clusters in different stages of neurogenic cells of young and old groups. ***P < 0.01. (D) Left, pie plot showing overlapped genes between cluster 3 DEGs and aging-related downregulated DEGs of TAPC in the monkey hippocampus. Right, bar plot showing enriched GO terms of the overlapped genes listed on the left. (E) Left, pie plot showing overlapped genes between cluster 5 DEGs and aging-related downregulated DEGs of neurons in the monkey hippocampus. Right, bar plot showing enriched GO terms of the overlapped genes listed on the left. (F) Network plot showing transcriptional regulators of aging-related DEGs in NSC and TAPC in the monkey hippocampus. Node size indicates the number of target genes. Outer circle of the node indicates the proportion of up regulated (red) and down regulated (blue) target genes regulated by corresponding transcriptional regulators. (G) Bar plot showing GO terms of target genes of *ZMAT4*. Red, upregulation; blue, downregulation. (H) Bar plot showing GO terms of target genes of *NR2F2*. Red, upregulation; blue, downregulation. (I) Violin plots showing expression levels of *NFIA*, *NFIB* and *NFIX* in TAPC in the monkey hippocampus from young and old groups. (J) Spearman’s correlations between gene set score of cluster 3 DEGs and the expression levels of *NFIA*, *NFIB* and *NFIX* in TAPC of the monkey hippocampus. (K) Density plot showing gene set scores of genes related to canonical Wnt signaling pathway in TAPC of the monkey hippocampus from young and old groups. (L) Heatmap showing the expression changes of aging-related DEGs associated with canonical Wnt signaling in TAPC of the monkey hippocampus from young and old groups.

To determine the molecular disruptions underlying impaired neurogenesis with age, we first inferred the differentiation trajectories of NSC in the hippocampal neurogenesis, starting from NSCs, through sequential and stepwise developmental progenitor cells, immature neurons, and finally to mature neurons, as indicated by pseudotime analysis (Figs. 5A and S3A), and observed no obvious difference of cell-type distribution along the trajectories between the young and old groups (Fig. 5A). Also, we established the underlying molecular cascades with respect to the pseudotime. For example, the differential expression of representative maker genes reflected the transition from quiescence to activation of NSCs, then proliferation, and differentiation into neurons (Fig. S3B). After clustering of stage-specific gene expression along the pseudotime, we obtained five distinct clusters of expression profiles and analyzed their corresponding enriched GO terms (Fig. 5B and Table S4). For example, cluster 3 defined genes progressively downregulated with the trajectories, which were enriched in pathways regulating “establishment of maintenance of cell polarity” (Fig. 5B). By contrast, cluster 5 defined genes progressively upregulated along the trajectories, and signature genes were involved in “trans-synaptic signaling” and “regulation of ion transport” (Fig. 5B).

Next, to investigate age-related alterations along the trajectories during neurogenesis, we compared the expression profiles of cluster-specific genes between the young and the old (Fig. 5C). We observed significant downregulations of cluster 3-specific genes in aged TAPC and of cluster 5-specific genes in aged neurons (Fig. 5C). Notably, by joint analysis of cluster-specific DEGs and age-related DEGs, we found 16 downregulated genes enriched for “cell polarity” and “cell division” in TAPC *(NFIA*, *MAML2*, etc.) (Fig. 5D), reminiscent of the reduced proportion of S-phase TAPC in the aged hippocampus and decreased DCX-positive progenitor cells in the DG region (Figs. 1E and S3C). And 14 downregulated genes shared between cluster 5-specific DEGs and aged neuron-specific DEGs (*ARPP21*, *NRG1*, etc.) were enriched for “cation transmembrane transport” (Fig. 5E), indicating the possibility of dysfunction in neurons of the aged hippocampus. Collectively, these results suggested aberrant neurogenesis in the aged hippocampus, including compromised TAPC proliferation at the initial stage and impaired neuron functions at the late stage of neurogenesis.

Then we set out to explore the transcriptional regulatory network governing age-associated DEGs of neuronal lineage cells. Of note, the results revealed a panel of key transcription factors (TFs), such as *ZMAT4* (Zinc Finger Matrin-Type 4), *NR2F2* (Nuclear Receptor Subfamily 2 Group F Member 2), *NFIX* (Nuclear Factor I X), etc. in TAPC and *KAT2A* (Lysine Acetyltransferase 2A) in NSC (Fig. 5F and Table S5), which regulated the expression of a set of genes associated with memory and learning (Fig. S3D). Specifically, GO enrichment analysis of genes targeted by *ZMAT4* and *NR2F2,* respectively, pinpointed that these two major hub TFs regulated a series of events involved in neurogenesis and neuronal synaptic signaling (Fig. 5G and 5H). Interestingly, we also noticed a consistent downregulation of Nuclear Factor I (NFI) family members including *NFIA*, *NFIB*, *NFIX* in aged TAPC (Fig. 5D, 5F and 5I). Combined with a correlation analysis, we demonstrated a positive correlation of NFIX family members with cluster 3-specific genes (Fig. 5J), which are highly expressed at the early stage of the neurogenesis trajectory. This suggests a prominent role of NFI family TFs in the initiation of TAPC differentiation, whose downregulation may contribute to an aberrant transcriptional regulatory network and compromise the initial stages of neurogenesis. Other key TFs, such as *TCF7L1* and *TCF12* (Fig. 5F), were core TFs involved in the Wnt signaling pathway. Therefore, we performed the gene set score analysis for the canonical Wnt signaling pathway (Fig. 5K). As expected, we observed a decrease of the gene set score in the aged samples, reflected by an upregulation of inhibitory genes in Wnt signaling including *ANKRD6* and *HECW1*, as well as a downregulation of core activating genes such as *CTNND2* (Fig. 5L). As Wnt signaling is essential for NSC activation and TAPC differentiation (Bengoa-Vergniory and Kypta, 2015), its dysregulation further supported the compromised neurogenesis during hippocampal aging.

Altogether, dysregulation at multiple steps along the neurogenesis trajectory of the aged hippocampus, especially at the initial stage of neurogenesis and at the later stage of synaptic function, constituted one of the major alterations in the aged NHP hippocampus and may contribute to impaired cognition.

### Identification of elevated neuroinflammation in aged NHP hippocampus

**Figure 6:**
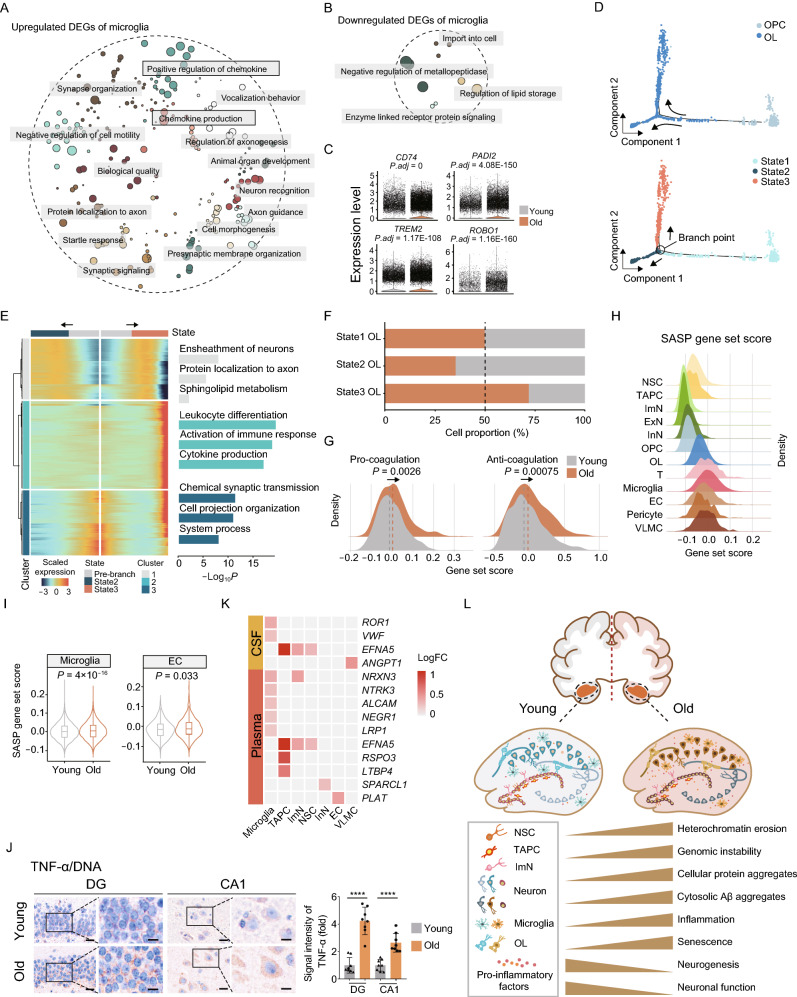
**Elevated inflammation with age in the monkey hippocampus**. (A) Dot plot showing GO terms of aging-related upregulated DEGs in microglia of the monkey hippocampus. Each dot indicates a GO term and similar entries were clustered together. (B) Dot plot showing GO terms of aging-related downregulated DEGs in microglia of the monkey hippocampus. Each dot indicates a GO term and similar entries were clustered together. (C) Violin plots showing expression levels of indicated genes in microglia of the monkey hippocampus from young and old. (D) Pseudotime analysis of OPC and OL in the monkey hippocampus. Cells are colored by the cell types (top) and the states (below). The arrows indicate the directions of differentiation trajectories. (E) Heatmap showing the expression profiles along the pseudotime of top 500 DEGs (q value < 1 × 10^−4^) in OL and OPC, which were then divided into three clusters with the expression pattern and enriched GO terms of the corresponding cluster represented on the right. (F) Bar plot showing the proportions of different states of OL in the hippocampus from young and old groups. (G) Density plot showing gene set scores of pro-coagulation and anti-coagulation genes in EC of the monkey hippocampus from young and old groups. (H) Density plot showing gene set scores of senescence-associated secretory phenotype (SASP) genes in different cell types in the monkey hippocampus. (I) Violin plots showing increasing SASP gene set scores in microglia and EC in the monkey hippocampus from young and old groups. (J) Immunohistochemical staining of TNF-α (brown) and counterstaining of cell nuclei by hematoxylin solution (blue) in the hippocampus from young and old monkeys. Representative images are shown on the left; signal intensity of TNF-α is quantified as fold changes in the old DG and CA1 regions vs. in young counterparts, shown as means ± SEM on the right. Scale bars, 20 μm and 10 μm (zoomed-in images). Young, *n* = 8; old, *n* = 8 monkeys. ****P < 0.0001. (K) Heatmap showing aging-related DEGs and their age-associated protein products in human cerebrospinal fluid (CSF) and plasma in different cell types in the monkey hippocampus. (L) A schematic illustration showing the phenotypic and transcriptomic signatures of NHP hippocampal aging.

In addition to intrinsic changes, neurogenesis is profoundly influenced by the neurogenic niche, which is comprised of microglia, oligodendrocytes, neurovasculature, and the systemic cytokine and chemokine environment (Kempermann et al., 2015). It has been reported that neuroinflammation is a known negative regulator of adult hippocampal neurogenesis (Fan et al., 2017; Jin et al., 2021; Leng and Edison, 2021), in which age-induced microglia activation and pro-inflammatory factors play a central role (Leng and Edison, 2021). Through analysis of age-related DEGs, we observed that upregulated genes were enriched for “regulation of chemokine” and “chemokine production” in the aged cells (Fig. 6A and 6B). Moreover, a panel of genes related to microglia activation, i.e., *CD74*, *TREM2*, *PADI2*, *ROBO1*, were upregulated in the aged microglia compared to their younger counterparts (Fig. 6C). For example, the established marker for activated M1 microglia CD74 acts as a cell-surface receptor for macrophage migration inhibitory factor (MIF) and a multifunctional trigger for the inflammatory response (Hwang et al., 2017; Su et al., 2017). TREM2 contributes to the age-related microglial activation, the phagocytic oxidative burst, and the loss of neurons with possible detrimental effects during physiological aging, and were recently found to increase the risk for developing AD (Leyns et al., 2017; Linnartz-Gerlach et al., 2019). PADI2 is known to promote the production of pro-inflammatory factors IL-1β, IL-6, and TNF-α in macrophages, and has been implicated in chronic inflammatory diseases (Wang et al., 2017; Yu et al., 2020). SLIT2-ROBO1 signaling also associates with the regulation of genes involved in pro-inflammation (Geutskens et al., 2010; Tiensuu et al., 2019).

Oligodendrocyte, a type of neuroglia that produces myelin, an insulating sheath on the axons of nerve fibers, constitutes an important part of the microenvironment for neurogenesis (Marques et al., 2016). We next traced the aging-related molecular changes along their developmental trajectory and reconstructed the sequential developmental steps by pseudotime analysis (Figs. 6D and S4A). Differential expression of representative marker genes demonstrated the transition from OPC to OL (Fig. S4B). After clustering of stage-specific gene expression along the pseudotime, we obtained three distinct clusters of expression profiles and their corresponding gene sets (Fig. 6E and Table S4). Through GO term analysis, we found that the gene set 1 was enriched for genes responsible for “ensheathment of neurons”, gene set 2 was enriched for genes involved in “leukocyte differentiation” and “activation of immune response”, and gene set 3 was enriched for genes regulating “chemical synaptic transmission” (Fig. 6E). Interestingly, gene set 2 is specifically expressed in an OL subtype (state 3 OL), whose population expanded at the advanced age (Figs. 6F and S4C). Collectively, these findings suggest an age-associated activation of inflammation and consequent promotion of a pro-inflammatory microenvironment impairing neurogenesis.

The neurovasculature is a critical node connecting the neurogenic niche with the systemic environment, whose dysregulation has been proposed to impair neurogenesis. As aforementioned, we found increased zymogen activation in the aged ECs (Fig. 4D), including PLAT, a plasminogen activator required for fibrinolysis (Kruithof and Dunoyer-Geindre, 2014), whose upregulation may result in the formation of hemolysis in hippocampus. However, when we evaluated the expression of coagulation-related genes in ECs, we found that gene set scores of both pro-coagulation and anti-coagulation pathways were higher in aged ECs compared with those in their young counterparts (Fig. 6G), implicating the imbalance and dysregulation of coagulation and hemolysis. As coagulation and hyperfibrinolysis are closely related to disruption of blood-brain barrier permeability (Marcos-Contreras et al., 2016; Gust et al., 2017), our results suggest a plausible blood-brain barrier dysfunction involved in hippocampal aging.

We observed increased senescent cells in various regions of the aged hippocampus (Fig. 1D). As senescence-associated secretory phenotype (SASP) is a common feature of senescent cells and usually contributes to a low-grade inflammatory state (Baker and Petersen, 2018), we next asked if the neurogenic niche in the aged hippocampus presents elevated SASP and systemic pro-inflammatory environment. Accordingly, we noticed that various niche cells exhibited much higher gene set scores for SASP overall (Fig. 6H). Especially, for microglia and EC, the two cell types that constitute the blood-brain barrier (Vanlandewijck et al., 2018), we found elevated SASP scores with age (Figs. 6I and S4D). Our findings may reflect a rise in age-related inflammation responses observed in these two cell types and a plausible destruction of the hippocampal blood-brain barrier with age (Sweeney et al., 2018; Yang et al., 2020). Consistently, we discovered an increase in pro-inflammatory factors (Tilstra et al., 2011), represented by age-related elevation of tumor necrosis factor-alpha (TNF-α)-positive cells (Figs. 6J and S4E). Moreover, we used joint analysis of the upregulated genes in the NHP hippocampus with proteomic data from aged human cerebrospinal fluid (CSF) or plasma samples to pinpoint the conserved alteration in protein products in primates (Baird et al., 2012; Tanaka et al., 2018). We found that microglia harbor the highest number of conserved changes, including *ROR1* and *VWF* in the aged CSF, and *NRXN3*, *NTRK3* and *ALCAM1* in the plasma from the elderlies, respectively, providing the potential for microglia-secreted factors to serve as circulatory hallmarks in the elderlies (Fig. 6K).

To sum up, these findings indicated a pro-inflammatory neurogenic niche and underlying molecular features during hippocampal aging, suggesting the formation of a deleterious microenvironment in the primate hippocampus at an advanced age, which may exacerbate age-related neurodegeneration and facilitate the progression of neurodegenerative diseases.

## Discussion

To date, the cellular and molecular mechanisms underlying primate hippocampal aging have not been determined. In this study, we systemically depicted the aging-associated phenotypes, and found that NHP hippocampus underwent extensive aging-related damages during physiological aging without obvious structural disorganization. Next, we established the first primate single-nucleus transcriptomic roadmap of hippocampal aging. According to their unique transcriptional signatures, we identified 12 distinct cell types, comprised of the neurogenesis lineage, oligodendrocyte lineage, and the niche cells. Aging-related DEG analysis unraveled that TAPC and microglia were the most susceptible cell types to aging as they manifested the most DEGs, including genes annotated as high-risk genes for neurodegenerative diseases. In-depth analysis of the dynamic gene-expression signatures of the stepwise neurogenesis trajectory revealed the impaired TAPC division and compromised neuronal function as prominent aging features. The elevated pro-inflammatory response was observed in the aged microglia, OL and EC, which may trigger the formation of an inflammaging microenvironment in the primate hippocampus. Furthermore, dysregulation of coagulation-regulating genes in aged EC suggested perturbance of the blood-brain barrier integrity as a potential factor associated with hippocampal degeneration. In summary, our analyses provided a wealth of resources for understanding the aging phenotypic changes and the underlying mechanisms of the primate hippocampal aging, and shed light on the development of novel diagnostic and therapeutic interventions against age-related neurodegenerative disorders (Fig. 6L).

Age-associated impairments of neurogenesis in DG of the hippocampus are associated with various neurodegenerative disorders (Morrison and Baxter, 2012; Fan et al., 2017; Hou et al., 2019), making hippocampus a crucial and attractive subject for both fundamental research and translational neurobiology. As a highly heterogeneous tissue, the complexity of hippocampal aging needs to be unveiled at the single-cell level. A pioneering study at single-cell resolution has been carried out in the mouse hippocampus specifically for the neurogenic niche, thus only focusing on the non-neuronal cell types. They focused more on the abundance of different cell types rather than their molecular profiles due to the limitation of available high-quality single cell transcriptomes in this early study (Artegiani et al., 2017). On the other hand, a following study supporting age-associated alterations in gene expression of neuronal cells has been reported for the neural stem/progenitor cell (NSPC) subpopulations by single-cell RNA-seq in the mouse brain, although it focused on NSPC localized in the subependymal/subventricular zone (SEZ/SVZ) of the forebrain, rather than those in the SGZ of the hippocampus (Shi et al., 2018). Yet another very recent study reported that NSC subpopulations exhibited aging-related asynchronous depletion accompanied by increased quiescence, and revealed a set of molecular hallmarks of aging in mouse hippocampus via single-cell transcriptomic analysis (Ibrayeva et al., 2021). All these previous studies advanced our understanding of age-related neurodegeneration, although we need to be cautious applying it to human diseases due to the drastic difference in physiological structure and functions between mouse and primate brains (Herculano-Houzel, 2009; Zhang et al., 2018; Zhong et al., 2020). Therefore, a high-resolution and comprehensive study to help understand age-related molecular changes in the primate hippocampus is of pressing demand.

With recent advances of snRNA-seq technologies (He et al., 2020; Ma et al., 2020a, b), we established a comprehensive single-nucleus transcriptomic atlas of monkey hippocampal aging, which provides extensive resources for the illustration of age-related molecular signatures at the single-cell level. Overall, we have captured a broad spectrum of cell types in the hippocampus, including the neurogenic lineage cells, oligodendrocyte lineage cells, microglia, and other niche cells. While our study failed to capture a cell cluster with the astrocyte signature, it may be attributed to the technological limitation in enriching astrocytes with the current procedures. One possibility is that astrocyte and NSC shared some common marker genes, implying that the NSC population may contain some astrocyte, which was consistent with the previous studies (Artegiani et al., 2017; Dulken et al., 2019). Another technical restriction that snRNA-seq only sequences the pre-mRNA in the nucleus with relatively low sequencing depth may also limit the comprehensiveness and dynamic range of detection, especially for mature neurons with dendrites and axons. In this context, spatial transcriptomic analysis, a newly developed technology (Stahl et al., 2016; Chen et al., 2020), may provide a potential resolution for measuring transcriptional profiles of previously undetectable cells.

Neurogenesis abnormality can lead to compromised neuron regeneration upon injury, along with progressive neurodegeneration and loss of memory in physiological aging (Wyss-Coray, 2016). Since the old hippocampi in the present study were still disease-free, an overall change in their major cell types were not yet presented, nor were the consequent alteration in its structure. Although we did observe reduced DCX^+^ TAPC and increased pro-inflammation OL in the elderly, not excluding the possibility of changes that may be revealed by future studies to classify hippocampal cells into finer subpopulations. But more importantly, we found significant remodeling in the transcriptomes of various cell types, reflecting dysregulation at multiple steps along the developmental trajectories of the neurogenic lineage cells in the aged NHP hippocampus, i.e., at the early onset of neurogenesis and at the later synaptic transmission stage. Especially, we found age-associated downregulation of NFI family members during the initiation of TAPC differentiation, which are import transcription factors in multiple organ development (Harris et al., 2015; Zenker et al., 2019). In line with our observations, recent studies demonstrated that knockout mice of either *Nfia*, *Nfib*, or *Nfix* displayed shared brain defects, including megalencephaly, dysgenesis of the corpus callosum, and a severely malformed hippocampus with reduced DG volume (Zenker et al., 2019). It was further supported by human phenotypes caused by the haploinsufficiency of *NFIA*, *NFIB*, and *NFIX* (Zenker et al., 2019), suggesting a common but not redundant role of these NFI members in brain development. Together with our findings, the function of these NFI members now extends to the protection of cognition during aging, providing potential targets to intervene disease progression in the nervous system.

On the other hand, neuroinflammation has also been documented for a crucial role in the pathogenesis of chronic neurodegenerative diseases, such as the progressive loss of memory and development of AD (Ransohoff, 2016; Leng and Edison, 2021). Notably, microglial cells are the primary players in neuroinflammation among the innate immune cells (Ransohoff, 2016). In line with previous observations, our single-nucleus transcriptomic atlas unraveled the upregulation of several pro-inflammatory genes, such as *CD74*, which can mediate the MIF signaling and promote the inflammatory response (Bryan et al., 2008), as well as the canonical pro-inflammatory factors TNF-α in the aged microglia. Of note, TNF-α has been reported to promote necroptosis mediated by receptor-interacting protein kinase 1 (RIPK1), RIPK3, and MLKL (Ofengeim and Yuan, 2013), which can robustly elicit the cascade of the inflammation responses, further magnifying the deleterious pro-inflammatory microenvironment that mediates the pathogenesis of various neurodegenerative diseases (Yuan et al., 2019). Interestingly, a recent study has suggested that microglia activated by Aβ deposition could produce neurotoxic cytokines and chemokines, including TNF, IL-6, IL-1β and CCL2, which subsequently cause neuronal dysfunction and death (Leng and Edison, 2021). Consistently, we observed elevated cytosolic Aβ deposition and accumulation of aggregates in the aged hippocampus, further supporting a potential connection between pathological protein aggregates and neuroinflammation in primate hippocampal aging. However, whether and how microglia-mediated age-related neuroinflammation suppress TAPC division and synaptic plasticity remain to be investigated in the future.

To our knowledge, we provided the first evidence that the NHP hippocampus underwent age-dependent genomic and epigenomic alterations, characterized by increased DNA damage, aggravated cytosolic dsDNA release from the nucleus, decondensed heterochromatin, and consequent derepression of heterochromatin-enriched LINE1 retrotransposon (LINE1). Emerging evidence indicates that destability of heterochromatin is a major driver for accelerated stem cell aging, and that consolidation of heterochromatin can rejuvenate aged stem cells (Zhang et al., 2015, 2020b; Deng et al., 2019; Bi et al., 2020; Hu et al., 2020; Diao et al., 2021; Liang et al., 2021). We hypothesized that the age-associated perturbation of the nuclear envelope may result in the leakage of the dsDNA and transcripts of derepressed repetitive sequences (Dou et al., 2015; Zhang et al., 2015, 2020b; Buchwalter et al., 2019; De Cecco et al., 2019; Deng et al., 2019; Geng et al., 2019; Simon et al., 2019; Bi et al., 2020; Hu et al., 2020; Diao et al., 2021; Liang et al., 2021; Liu et al., 2021), thus elicit consequent inflammation responses as observed in the aged NHP hippocampus. Consistently, it has been reported that nuclear envelope impairment is tightly associated with aging and neurodegenerative diseases, such as AD and PD (Liu et al., 2012; Frost, 2016; Bedrosian et al., 2021; Bin Imtiaz et al., 2021). When dsDNA accumulates in the cytoplasm, it usually triggers the cGAS-STING signaling pathway and subsequently activates the innate immune responses (Volkman and Stetson, 2014; Bi et al., 2020; Zhang et al., 2020b), which is frequently observed in senescent stem cells and a variety of age-related disorders as age-associated inflammation (De Cecco et al., 2019; Geng et al., 2019; Simon et al., 2019; Bi et al., 2020). Our findings propose a yet-to-be-explored link between (epi)genomic instability and elevated neuroinflammation in primate hippocampal aging.

All in all, by constructing the single-cell resolution transcriptomic atlas of primate hippocampal aging, we aimed to provide a deeper understanding of physiological aging of the primate hippocampus and its pathological relevance. We identified molecular features corresponding to (epi)genomic instability and accumulated damages, elevated neuroinflammation, compromised functions in specific cell types in the old hippocampus, which contribute collectively to the impaired ability for neuronal regeneration, thus enabling the identification of potential diagnostic biomarkers and therapeutic targets for neurodegenerative diseases associated with hippocampal aging.

## Materials and methods

### Ethical statement

This study was conducted following the guidelines for the Ethical Treatment of Non-Human Primates and was approved by the Institutional Animal Care and Use Committee of the Institute of Zoology (Chinese Academy of Sciences). All animal experiment procedures were performed by certified veterinarians, complying with laws governing animal research.

### Hippocampus tissue collection

Brain tissues from eight young (4–6 years old) and eight aged (18–21 years old) cynomolgus monkeys (*Macaca fascicularis*) were harvested as previously described (Li et al., 2020; Wang et al., 2020a, b; Zhang et al., 2020c). The monkeys were the same ones sacrificed in the aforementioned studies (Li et al., 2020; Wang et al., 2020a; Zhang et al., 2020c). After the perfusion was completed and the tissue turned white, right hemisphere of each brain was fixed in 4% paraformaldehyde for histological analyses. Left hemisphere of each brain was separated according to brain regions and was frozen in liquid nitrogen. The hippocampus is located on the medial temporal lobe. We carefully picked out the hippocampus on ice, divided them into pieces in order, placed them in a cryotube, and stored them in liquid nitrogen (During the collection of frozen tissue, the hippocampus from a young female individual was excluded due to limited technical experience at the beginning of the experiment).

### Nissl staining

Nissl staining was performed as previously described (Boldrini et al., 2018). After the hippocampus tissues fixed in 4% PFA are dehydrated in a series of graded alcohols (70%–100%), the tissues were embedded in paraffin and sliced to a thickness of 5 μm using a rotary microtome. The sections were placed on a microscope slide, dried at 56 °C for 2 h, and stored at room temperature (RT). For Nissl staining, sections were deparaffinized and rehydrated using xylene and graded alcohols (100%–70%), and then rinsed in distilled water. Tissue sections were incubated with Nissl Staining Solution (Beyotime, China) at RT for 10 min, and washed with distilled water and 70% ethanol twice. Sections were quickly dehydrated in 95% ethanol and 100% ethanol, cleared in xylene, and mounted with a resinous mounting medium.

### SA-β-Gal staining

SA-β-Gal staining was performed using a previously published protocol (Debacq-Chainiaux et al., 2009; Chow et al., 2019; Ma et al., 2020a, 2020b). In brief, the hippocampal tissue stored in the cryotube was fixed and dehydrated in a cold mixture of 75% ethanol and 4% paraformaldehyde at 4 °C shaking for 3 h, then in 4% paraformaldehyde at 4 °C shaking overnight, followed by shaking in 5% sucrose at 4 °C for 3 h and then in 30% sucrose at 4 °C shaking overnight or until the tissue shrinked completely. OCT-embedded, dehydrated hippocampus tissues were cryosectioned at a thickness of 10 μm with a Leica CM3050S cryomicrotome, collected on Superfrost Plus microslides (VWR) and stored at −80 °C until use. For SA-β-Gal staining, sections were thawed at RT and rinsed in PBS, fixed in 2% formaldehyde and 0.2% glutaraldehyde at RT for 5 min and stained with freshly prepared staining solution at 37 °C (X-gal was purchased from Amresco; all the other reagents were from Sigma-Aldrich). Images were taken with PerkinElmer Vectra Polaris, and the SA-β-Gal-positive areas were quantified using ImageJ.

### Aggresome staining

Aggresome staining was performed as previously described (Li et al., 2020). The paraffin section was deparaffinized and rehydrated using xylene and graded ethanol. Sections were permeabilized with 0.3% Triton X-100 in PBS for 7 min, incubated with the Aggresome dye (Proteostat® Protein aggregation assay, Enzo, ENZ-51023-KP002, 1:3,000 dilution in PBS) for 3 min and then destained in 1% acetic acid for 30 min. Next, sections were washed with PBS for 3 times (10 min each) at RT, and counterstained with Hoechst 33342 (Thermo Fisher Scientific). Zeiss 900 confocal system was used for aggresome staining microscopy.

### Immunofluorescence staining

Immunofluorescence staining was performed as described (Li et al., 2020; Wang et al., 2020a, b; Zhong et al., 2020). Briefly, paraffin-embedded sections were deparaffinized in xylene and rehydrated through gradient ethanol (100%–70%). After rinsing in distilled water, sections were microwaved in 10 mmol/L sodium citrate buffer (pH 6.0) 5 times for 3 min each. Upon cooling down to RT, sections were rinsed three times in PBS, permeabilized with 0.4% Triton X-100 in PBS for 2 h and rinsed again in PBS three times. Sections were then incubated with blocking buffer (10% donkey serum in PBS) at RT for 1 h, followed by incubation with primary antibodies overnight at 4 °C and fluorescence-labeled secondary antibodies at RT for 1 h. Nuclei were counterstained with Hoechst 33342 (Thermo Fisher Scientific) before the sections were mounted in VECTERSHIELD^®^ anti-fading mounting medium (Vector Laboratories, h-1000). Zeiss 900 confocal system was used for immunofluorescence microscopy. The antibodies used for immunofluorescence staining are as follows: anti-β-Amyloid (4G8) (Biolegend, 800701, 1:200), anti-H3K9me3 (Abcam, ab8898, 1:500), anti-LINE1 ORF2p (Abcam, ab106004, 1:200), anti-dsDNA (Santa Cruz Biotechnology, sc-58749, 1:500). Secondary antibodies used were the following: donkey anti rabbit-AF488 (Thermo Fisher (1:500)), donkey anti mouse-AF488 (Thermo Fisher (1:500)), donkey anti chicken-AF488 (Thermo Fisher (1:500)).

### Immunohistochemistry staining

Immunohistochemistry staining was performed as previously described (Li et al., 2020; Ma et al., 2020b). Briefly, the paraffin-embedded sections were deparaffinized and rehydrated using xylene and graded ethanol. Antigen retrieval was performed by steaming in citrate buffer 5 times for 3 min each. After being cooled down to RT, sections were rinsed three times in PBS, permeabilized with 0.4% Triton X-100 in PBS for 2 h and rinsed again in PBS three times. And then sections were incubated with 3% H_2_O_2_ for 10 min to inactivate endogenous peroxidase. Sections were then blocked with 10% donkey serum in PBS for 1 h and incubated with primary antibodies at 4 °C overnight. The next day, sections were incubated with HRP-conjugated secondary antibodies at RT for 1 h. Sections were performed using the DAB Staining Kit (ZSGB-BIO, ZLI-9018) according to the manufacturer’s instructions. Finally, sections were dehydrated in a series of graded alcohols (50%, 70%, 80%, 90%, 100%, and 100%) and xylene before being mounted in the neutral resinous mounting medium. The antibodies used for immunohistochemistry staining are as follows: anti-DCX (Abcam, ab18723, 1:200), anti-β-Amyloid (1-40) (Cell Signaling, 12990s, 1:400), anti-γH2A.X (Millipore, 05636, 1:100), anti-HP1γ (Cell Signaling, 2616s, 1:400), anti-TNF-α (Santa Cruz Biotechnology, sc-52746, 1:200). Images were taken with PerkinElmer Vectra Polaris.

### Nuclei isolation and snRNA-seq on the 10x Genomics platform

The protocol for the isolation of nuclei from frozen brain tissues was adapted from Krishnaswami et al. (2016) with minor modifications (Ma et al., 2020a, b). All procedures were carried out on ice or at 4 °C. Nuclei isolation media 1 and 2 (NIM1 and NIM2) and homogenization buffer were pre-chilled on ice. NIM1 contained 250 mmol/L sucrose, 25 mmol/L KCl, 5 mmol/L MgCl_2_, 10 mmol/L Tris buffer (pH 8.0) in nuclease-free water. NIM2 was the same as NIM1 except for the addition of 1 μmol/L DTT and 1× protease inhibitor. Homogenization buffer combined the following reagents, including NIM2, 0.4 U/μL RNaseIn, 0.2 U/µL Superasin, 0.1% Triton X-100, 1 μmol/L propidium iodide (PI), and 10 ng/mL Hoechst 33342. The Dounce homogenizer and pestles were pre-chilled on ice. Frozen brain tissues were homogenized with strokes of the pestle in 1 mL pre-chilled homogenization buffer. Next, put the tubes into dounce homogenizer, and grind for 60 hz, 60 s, 3 times. Then the homogenized tissue was filtered through a 40-μm cell strainer (BD Falcon), and 500 µL supernatant was collected. The nuclei were collected by centrifugation at 1000 ×*g* for 8 min at 4 °C. Samples were filtered through a 40-μm cell strainer, centrifuged at 1000 ×g for 8 min at 4 °C, and resuspended in PBS supplemented with 0.3% BSA, 0.4 U/μL RNaseIn and 0.2 U/μL Superasin. Nuclei were sorted by staining with both Hoechst 33342 and PI using fluorescence-activated cell sorting (FACS) (BD Influx) and counted with a dual-fluorescence cell counter (Luna-FLTM, Logos Biosystems). Mononuclear capture was conducted with a 10x Genomics single-cell 3’ system. Approximately 8,000 nuclei were captured for each sample following the standard 10x capture and library preparation protocol (10x Genomics) and then sequenced on a NovaSeq 6000 sequencing platform (Illumina, 20012866).

### Processing and quality control of snRNA-seq data

Raw sequencing reads were aligned to the pre-mRNA reference (Ensemble, Macaca_fascicularis_5.0) and counted using Cell Ranger (version 3.1.0) with the default parameters. SoupX (version 1.4.8) was applied to every sample to remove ambient RNA bias (Young and Behjati, 2020). Seurat (version 3.1.3) object of each sample was constructed from decontaminated matrix and cells with genes fewer than 200 or mitochondrial ratio more than 2.5% were discarded (Butler et al., 2018). Doublet detection was performed with DoubletFinder (version 2.0.2) (McGinnis et al., 2019). After sample integration and clustering as mentioned below, clusters lacking specific marker genes and with relatively low gene content and high mitochondrial ratio were also discarded.

### Integration, clustering, and identification of cell types

Dataset of each sample was normalized using the “SCTransform” function of Seurat. Features and anchors for downstream integration were selected with corresponding pipeline according to the SCTransform method, ensuring that calculation was based on all necessary Pearson residuals. After data integration and scaling, principal component analysis was applied with the “RunPCA” function and appropriate principal components were selected for the following analysis. Dimensionality was reduced with the “RunUMAP” function. “FindNeighbors” and “FindClusters” functions were applied to perform clustering. Marker genes for each cluster were identified by the “FindAllMarkers” function with the cutoff of adjusted *P*-values < 0.05 and |logFC| > 1 (Table S1). Canonical marker genes were used to identify cell types.

### Transcriptional noise analysis

Transcriptional noise was estimated as previously described (Angelidis et al., 2019). In brief, equal number of cells of each cell type were used between the young and old groups. All genes were ordered by their expression levels, and those with the top 10% and bottom 10% expression levels were excluded. Transcriptional noise at the cell level was then calculated as the euclidean distances between cells with the mean value of the corresponding cell type. For transcriptional noise at the sample level, the euclidean distances between young and old samples were averaged for each cell type.

### Age-related differential gene expression analysis

Differentially expressed genes (DEGs) were identified with the “FindMarkers” function of Seurat in every cell type between young and old samples using the Wilcoxon test with the threshold of adjusted *P*-values < 0.05 and |logFC| > 0.25 (Table S2).

### Transcriptional regulatory network analysis

Transcriptional regulatory network analysis was conducted using SCENIC workflow (version 1.1.2.2) with default parameters based on hg19 database from RcisTarget (version 1.6.0) (Aibar et al., 2017). For cell-type-specific transcription regulatory network, only marker genes were used and calculation was performed in each cell type. And cell types with cell more than 20,000 (microglia and OL) were subsampled to 20,000 cells. For age-related transcriptional regulatory network, only age-related DEGs were used as input for transcriptional regulator inferring and all selected cell types were calculated together. Obtained transcription regulatory network was visualized by Cytoscape (version 3.8.2) (Shannon et al., 2003).

### GO term analysis

FunSet (http://funset.uno/) and Metascape were used to perform GO term (http://metascape.org/gp/index.html) (version 3.5) (Zhou et al., 2019). Results were further visualized with the ggplot2 R package (https://ggplot2.tidyverse.org/) (version 3.2.1).

### Pseudotime analysis

Pseudotime analysis was performed with using the Monocle2 R package (Trapnell et al., 2014). Top 200 highly variable genes from the “FindMarkers” function of Seurat package were used as ordering genes with the threshold of adjusted *P*-value < 0.05 and |logFC| > 1. DDRTree dimensionality reduction method was applied to construct the trajectory that was plotted in two-dimensional space. Differentiation-related DEGs were obtain with a cutoff of q value < 1 × 10^−4^.

### Cell-cell communication analysis

Cell-cell communication analysis was performed using the CellPhoneDB (version 1.1.0) (Efremova et al., 2020). Only receptors and ligands expressed in more than 10% cells of any cell type from either young or old samples were further evaluated. Only those with a *P*-value < 0.01 were used for the prediction of cell-cell communication between any two cell types.

### Gene set score analysis

Gene sets related to aging-related diseases were obtained from database DisGeNET (https://www.disgenet.org/home/). Gene sets of AD and PD were generated by filtering with “diseaseName” (“Alzheimer’s disease” and “Parkinson disease”, respectively). Gene set of learning disorders consists of genes related to “Learning Disorders”, “Learning Disturbance” and “Learning Disabilities”, while that of memory disorders includes genes related to “Age-Related Memory Disorders”, “Memory Disorders”, “Memory impairment”, “Memory Disorder, Semantic”, “Memory Disorder, Spatial” and “Memory Loss”. Other gene sets used in this paper were acquired from GSEA (Subramanian et al., 2005). Genes related to pro-/anti-coagulation and SASP were manually curated from previous literatures (Hoppe and Dorner, 2012; Ma et al., 2020a, b; Gu et al., 2021). All gene sets involved in this study are summarized in Table S6. Gene set scores were acquired by analyzing the transcriptome of each input cell against aforementioned gene sets by the Seurat function “AddModuleScore”. Changes in the scores between young and old samples were analyzed using ggpubr R package via the Wilcoxon test (https://github.com/kassambara/ggpubr) (version 0.2.4).

### Statistical analysis

All experimental data were statistically analyzed using PRISM software (GraphPad 8 Software). Results were presented as mean ± SEM. Comparisons were conducted using the two-tailed Student’s *t*-test.

## Supplementary Information

Below is the link to the electronic supplementary material.

Supplementary material 1 (PDF 19704 kb)

Supplementary material 2 (XLSX 369 kb)

Supplementary material 3 (XLSX 108 kb)

Supplementary material 4 (XLSX 225 kb)

Supplementary material 5 (XLSX 28 kb)

Supplementary material 6 (XLSX 50 kb)

Supplementary material 7 (XLSX 25 kb)

## References

[CR1] Aging Atlas C (2021). Aging Atlas: a multi-omics database for aging biology. Nucleic Acids Res.

[CR2] Aibar S, González-Blas CB, Moerman T, Huynh-Thu VA, Imrichova H, Hulselmans G, Rambow F, Marine J-C, Geurts P, Aerts J (2017). SCENIC: single-cell regulatory network inference and clustering. Nat Methods.

[CR3] Aimone JB, Li Y, Lee SW, Clemenson GD, Deng W, Gage FH (2014). Regulation and function of adult neurogenesis: from genes to cognition. Physiol Rev.

[CR4] Angelidis I, Simon LM, Fernandez IE, Strunz M, Mayr CH, Greiffo FR, Tsitsiridis G, Ansari M, Graf E, Strom TM (2019). An atlas of the aging lung mapped by single cell transcriptomics and deep tissue proteomics. Nat Commun.

[CR5] Artegiani B, Lyubimova A, Muraro M, van Es JH, van Oudenaarden A, Clevers H (2017). A single-cell RNA sequencing study reveals cellular and molecular dynamics of the hippocampal neurogenic niche. Cell Rep.

[CR6] Baird GS, Nelson SK, Keeney TR, Stewart A, Williams S, Kraemer S, Peskind ER, Montine TJ (2012). Age-dependent changes in the cerebrospinal fluid proteome by slow off-rate modified aptamer array. Am J Pathol.

[CR7] Baker DJ, Petersen RC (2018). Cellular senescence in brain aging and neurodegenerative diseases: evidence and perspectives. J Clin Investig.

[CR8] Bedrosian TA, Houtman J, Eguiguren JS, Ghassemzadeh S, Rund N, Novaresi NM, Hu L, Parylak SL, Denli AM, Randolph-Moore L (2021). Lamin B1 decline underlies age-related loss of adult hippocampal neurogenesis. EMBO J.

[CR9] Bengoa-Vergniory N, Kypta RM (2015). Canonical and noncanonical Wnt signaling in neural stem/progenitor cells. Cell Mol Life Sci.

[CR10] Bi S, Liu Z, Wu Z, Wang Z, Liu X, Wang S, Ren J, Yao Y, Zhang W, Song M (2020). SIRT7 antagonizes human stem cell aging as a heterochromatin stabilizer. Protein Cell.

[CR11] Bin Imtiaz MK, Jaeger BN, Bottes S, Machado RAC, Vidmar M, Moore DL, Jessberger S (2021). Declining lamin B1 expression mediates age-dependent decreases of hippocampal stem cell activity. Cell Stem Cell.

[CR12] Boldrini M, Fulmore CA, Tartt AN, Simeon LR, Pavlova I, Poposka V, Rosoklija GB, Stankov A, Arango V, Dwork AJ (2018). Human hippocampal neurogenesis persists throughout aging. Cell Stem Cell.

[CR13] Brunk UT, Terman A (2002). The mitochondrial-lysosomal axis theory of aging. Eur J Biochem.

[CR14] Bryan KJ, Zhu X, Harris PL, Perry G, Castellani RJ, Smith MA, Casadesus G (2008). Expression of CD74 is increased in neurofibrillary tangles in Alzheimer’s disease. Mol Neurodegener.

[CR15] Buchwalter A, Kaneshiro JM, Hetzer MW (2019). Coaching from the sidelines: the nuclear periphery in genome regulation. Nat Rev Genet.

[CR16] Buckig A, Tikkanen R, Herzog V, Schmitz A (2002). Cytosolic and nuclear aggregation of the amyloid beta-peptide following its expression in the endoplasmic reticulum. Histochem Cell Biol.

[CR17] Butler A, Hoffman P, Smibert P, Papalexi E, Satija R (2018). Integrating single-cell transcriptomic data across different conditions, technologies, and species. Nat Biotechnol.

[CR18] Chen Y, Niu Y, Ji W (2012). Transgenic nonhuman primate models for human diseases: approaches and contributing factors. J Genet Genom.

[CR19] Chen Y, Niu Y, Ji W (2016). Genome editing in nonhuman primates: approach to generating human disease models. J Intern Med.

[CR20] Chen Y, Yu J, Niu Y, Qin D, Liu H, Li G, Hu Y, Wang J, Lu Y, Kang Y (2017). Modeling Rett syndrome using TALEN-edited MECP2 mutant cynomolgus monkeys. Cell.

[CR21] Chen WT, Lu A, Craessaerts K, Pavie B, Sala Frigerio C, Corthout N, Qian X, Lalakova J, Kuhnemund M, Voytyuk I (2020). Spatial transcriptomics and in situ sequencing to study Alzheimer’s disease. Cell.

[CR22] Chow HM, Shi M, Cheng A, Gao Y, Chen G, Song X, So RWL, Zhang J, Herrup K (2019). Age-related hyperinsulinemia leads to insulin resistance in neurons and cell-cycle-induced senescence. Nat Neurosci.

[CR23] Colman RJ (2018). Non-human primates as a model for aging. Biochim Biophys Acta Mol Basis Dis.

[CR24] Costa-Mattioli M, Walter P (2020). The integrated stress response: from mechanism to disease. Science.

[CR25] De Cecco M, Ito T, Petrashen AP, Elias AE, Skvir NJ, Criscione SW, Caligiana A, Brocculi G, Adney EM, Boeke JD (2019). L1 drives IFN in senescent cells and promotes age-associated inflammation. Nature.

[CR26] Debacq-Chainiaux F, Erusalimsky JD, Campisi J, Toussaint O (2009). Protocols to detect senescence-associated beta-galactosidase (SA-βgal) activity, a biomarker of senescent cells in culture and in vivo. Nat Protoc.

[CR27] Deng L, Ren R, Liu Z, Song M, Li J, Wu Z, Ren X, Fu L, Li W, Zhang W (2019). Stabilizing heterochromatin by DGCR8 alleviates senescence and osteoarthritis. Nat Commun.

[CR28] Diao Z, Ji Q, Wu Z, Zhang W, Cai Y, Wang Z, Hu J, Liu Z, Wang Q, Bi S (2021). SIRT3 consolidates heterochromatin and counteracts senescence. Nucleic Acids Res.

[CR29] Dou Z, Xu C, Donahue G, Shimi T, Pan JA, Zhu J, Ivanov A, Capell BC, Drake AM, Shah PP (2015). Autophagy mediates degradation of nuclear lamina. Nature.

[CR30] Dulken BW, Buckley MT, Navarro Negredo P, Saligrama N, Cayrol R, Leeman DS, George BM, Boutet SC, Hebestreit K, Pluvinage JV (2019). Single-cell analysis reveals T cell infiltration in old neurogenic niches. Nature.

[CR31] Efremova M, Vento-Tormo M, Teichmann SA, Vento-Tormo R (2020). Cell PhoneDB: inferring cell–cell communication from combined expression of multi-subunit ligand–receptor complexes. Nat Protoc.

[CR32] Encinas JM, Michurina TV, Peunova N, Park JH, Tordo J, Peterson DA, Fishell G, Koulakov A, Enikolopov G (2011). Division-coupled astrocytic differentiation and age-related depletion of neural stem cells in the adult hippocampus. Cell Stem Cell.

[CR33] Fan X, Wheatley EG, Villeda SA (2017). Mechanisms of hippocampal aging and the potential for rejuvenation. Annu Rev Neurosci.

[CR34] Frost B (2016). Alzheimer’s disease: an acquired neurodegenerative laminopathy. Nucleus.

[CR35] Geng L, Liu Z, Wang S, Sun S, Ma S, Liu X, Chan P, Sun L, Song M, Zhang W (2019). Low-dose quercetin positively regulates mouse healthspan. Protein Cell.

[CR36] Geutskens SB, Hordijk PL, van Hennik PB (2010). The chemorepellent Slit3 promotes monocyte migration. J Immunol.

[CR37] Giacobini E, Gold G (2013). Alzheimer disease therapy–moving from amyloid-beta to tau. Nat Rev Neurol.

[CR38] Gu SX, Tyagi T, Jain K, Gu VW, Lee SH, Hwa JM, Kwan JM, Krause DS, Lee AI, Halene S (2021). Thrombocytopathy and endotheliopathy: crucial contributors to COVID-19 thromboinflammation. Nat Rev Cardiol.

[CR39] Gust J, Hay KA, Hanafi LA, Li D, Myerson D, Gonzalez-Cuyar LF, Yeung C, Liles WC, Wurfel M, Lopez JA (2017). Endothelial activation and blood-brain barrier disruption in neurotoxicity after adoptive immunotherapy with CD19 CAR-T cells. Cancer Discov.

[CR40] Habib N, Li Y, Heidenreich M, Swiech L, Avraham-Davidi I, Trombetta JJ, Hession C, Zhang F, Regev A (2016). Div-Seq: single-nucleus RNA-Seq reveals dynamics of rare adult newborn neurons. Science.

[CR41] Harris L, Genovesi LA, Gronostajski RM, Wainwright BJ, Piper M (2015). Nuclear factor one transcription factors: divergent functions in developmental versus adult stem cell populations. Dev Dyn.

[CR42] He G, Luo W, Li P, Remmers C, Netzer WJ, Hendrick J, Bettayeb K, Flajolet M, Gorelick F, Wennogle LP (2010). Gamma-secretase activating protein is a therapeutic target for Alzheimer’s disease. Nature.

[CR43] He X, Memczak S, Qu J, Belmonte JCI, Liu GH (2020). Single-cell omics in ageing: a young and growing field. Nat Metab.

[CR44] Head D, Snyder AZ, Girton LE, Morris JC, Buckner RL (2005). Frontal-hippocampal double dissociation between normal aging and Alzheimer’s disease. Cereb Cortex.

[CR45] Herculano-Houzel S (2009). The human brain in numbers: a linearly scaled-up primate brain. Front Hum Neurosci.

[CR46] Hoppe B, Dorner T (2012). Coagulation and the fibrin network in rheumatic disease: a role beyond haemostasis. Nat Rev Rheumatol.

[CR47] Hou Y, Dan X, Babbar M, Wei Y, Hasselbalch SG, Croteau DL, Bohr VA (2019). Ageing as a risk factor for neurodegenerative disease. Nat Rev Neurol.

[CR48] Hu H, Ji Q, Song M, Ren J, Liu Z, Wang Z, Liu X, Yan K, Hu J, Jing Y (2020). ZKSCAN3 counteracts cellular senescence by stabilizing heterochromatin. Nucleic Acids Res.

[CR49] Hwang IK, Park JH, Lee TK, Kim DW, Yoo KY, Ahn JH, Kim YH, Cho JH, Kim YM, Won MH (2017). CD74-immunoreactive activated M1 microglia are shown late in the gerbil hippocampal CA1 region following transient cerebral ischemia. Mol Med Rep.

[CR50] Ibrayeva A, Bay M, Pu E, Jorg DJ, Peng L, Jun H, Zhang N, Aaron D, Lin C, Resler G (2021). Early stem cell aging in the mature brain. Cell Stem Cell.

[CR51] Jin WN, Shi K, He W, Sun JH, Van Kaer L, Shi FD, Liu Q (2021). Neuroblast senescence in the aged brain augments natural killer cell cytotoxicity leading to impaired neurogenesis and cognition. Nat Neurosci.

[CR52] Kempermann G, Song H, Gage FH (2015). Neurogenesis in the Adult Hippocampus. Cold Spring Harb Perspect Biol.

[CR53] Keren-Shaul H, Spinrad A, Weiner A, Matcovitch-Natan O, Dvir-Szternfeld R, Ulland TK, David E, Baruch K, Lara-Astaiso D, Toth B (2017). A unique microglia type associated with restricting development of Alzheimer’s disease. Cell.

[CR54] Krishnaswami SR, Grindberg RV, Novotny M, Venepally P, Lacar B, Bhutani K, Linker SB, Pham S, Erwin JA, Miller JA (2016). Using single nuclei for RNA-seq to capture the transcriptome of postmortem neurons. Nat Protoc.

[CR55] Kruithof EK, Dunoyer-Geindre S (2014). Human tissue-type plasminogen activator. Thromb Haemost.

[CR56] Kuhn HG, Toda T, Gage FH (2018). Adult hippocampal neurogenesis: a coming-of-age story. J Neurosci.

[CR57] Leng F, Edison P (2021). Neuroinflammation and microglial activation in Alzheimer disease: where do we go from here?. Nat Rev Neurol.

[CR58] Leuner B, Kozorovitskiy Y, Gross CG, Gould E (2007). Diminished adult neurogenesis in the marmoset brain precedes old age. Proc Natl Acad Sci USA.

[CR59] Leyns CEG, Ulrich JD, Finn MB, Stewart FR, Koscal LJ, Remolina Serrano J, Robinson GO, Anderson E, Colonna M, Holtzman DM (2017). TREM2 deficiency attenuates neuroinflammation and protects against neurodegeneration in a mouse model of tauopathy. Proc Natl Acad Sci USA.

[CR60] Li R, Lindholm K, Yang LB, Yue X, Citron M, Yan R, Beach T, Sue L, Sabbagh M, Cai H (2004). Amyloid beta peptide load is correlated with increased beta-secretase activity in sporadic Alzheimer’s disease patients. Proc Natl Acad Sci USA.

[CR61] Li D, Takeda N, Jain R, Manderfield LJ, Liu F, Li L, Anderson SA, Epstein JA (2015). Hopx distinguishes hippocampal from lateral ventricle neural stem cells. Stem Cell Res.

[CR62] Li J, Zheng Y, Yan P, Song M, Wang S, Sun L, Liu Z, Ma S, Belmonte JCI, Chan P (2020). A single-cell transcriptomic atlas of primate pancreatic islet aging. Natl Sci Rev.

[CR63] Liang C, Liu Z, Song M, Li W, Wu Z, Wang Z, Wang Q, Wang S, Yan K, Sun L (2021). Stabilization of heterochromatin by CLOCK promotes stem cell rejuvenation and cartilage regeneration. Cell Res.

[CR64] Linnartz-Gerlach B, Bodea LG, Klaus C, Ginolhac A, Halder R, Sinkkonen L, Walter J, Colonna M, Neumann H (2019). TREM2 triggers microglial density and age-related neuronal loss. Glia.

[CR65] Liu GH, Qu J, Suzuki K, Nivet E, Li M, Montserrat N, Yi F, Xu X, Ruiz S, Zhang W (2012). Progressive degeneration of human neural stem cells caused by pathogenic LRRK2. Nature.

[CR66] Liu X, Liu Z, Sun L, Ren J, Wu Z, Jiang X, Ji Q, Wang Q, Fan Y, Cai Y (2021). Resurrection of human endogenous retroviruses during aging reinforces senescence. bioRxiv.

[CR67] Lubbe SJ, Bustos B, Hu J, Krainc D, Joseph T, Hehir J, Tan M, Zhang W, Escott-Price V, Williams NM (2021). Assessing the relationship between monoallelic PRKN mutations and Parkinson’s risk. Human Mol Genet.

[CR68] Ma S, Sun S, Geng L, Song M, Wang W, Ye Y, Ji Q, Zou Z, Wang S, He X (2020). Caloric restriction reprograms the single-cell transcriptional landscape of rattus norvegicus aging. Cell.

[CR69] Ma S, Sun S, Li J, Fan Y, Qu J, Sun L, Wang S, Zhang Y, Yang S, Liu Z (2020). Single-cell transcriptomic atlas of primate cardiopulmonary aging. Cell Res.

[CR70] Malykhin NV, Bouchard TP, Camicioli R, Coupland NJ (2008). Aging hippocampus and amygdala. NeuroReport.

[CR71] Marcos-Contreras OA, Martinez de Lizarrondo S, Bardou I, Orset C, Pruvost M, Anfray A, Frigout Y, Hommet Y, Lebouvier L, Montaner J (2016). Hyperfibrinolysis increases blood-brain barrier permeability by a plasmin- and bradykinin-dependent mechanism. Blood.

[CR72] Marques S, Zeisel A, Codeluppi S, van Bruggen D, Mendanha Falcao A, Xiao L, Li H, Haring M, Hochgerner H, Romanov RA (2016). Oligodendrocyte heterogeneity in the mouse juvenile and adult central nervous system. Science.

[CR73] Martinelli P, Real FX (2019). Mouse models shed light on the SLIT/ROBO pathway in pancreatic development and cancer. Trends Cancer.

[CR74] Mauffrey P, Tchitchek N, Barroca V, Bemelmans A-P, Firlej V, Allory Y, Roméo P-H, Magnon C (2019). Progenitors from the central nervous system drive neurogenesis in cancer. Nature.

[CR75] McGinnis CS, Murrow LM, Gartner ZJ (2019). DoubletFinder: doublet detection in single-cell RNA sequencing data using artificial nearest neighbors. Cell Syst.

[CR76] Morrison JH, Baxter MG (2012). The ageing cortical synapse: hallmarks and implications for cognitive decline. Nat Rev Neurosci.

[CR77] Nakamura R, Nakamoto C, Obama H, Durward E, Nakamoto M (2012). Structure-function analysis of Nel, a thrombospondin-1-like glycoprotein involved in neural development and functions. J Biol Chem.

[CR78] Navarro Negredo P, Yeo RW, Brunet A (2020). Aging and rejuvenation of neural stem cells and their niches. Cell Stem Cell.

[CR79] Ofengeim D, Yuan J (2013). Regulation of RIP1 kinase signalling at the crossroads of inflammation and cell death. Nat Rev Mol Cell Biol.

[CR81] Ransohoff RM (2016). How neuroinflammation contributes to neurodegeneration. Science.

[CR82] Rivero O, Sich S, Popp S, Schmitt A, Franke B, Lesch KP (2013). Impact of the ADHD-susceptibility gene CDH13 on development and function of brain networks. Eur Neuropsychopharmacol.

[CR83] Shannon P, Markiel A, Ozier O, Baliga NS, Wang JT, Ramage D, Amin N, Schwikowski B, Ideker T (2003). Cytoscape: a software environment for integrated models of biomolecular interaction networks. Genome Res.

[CR84] Shi Z, Geng Y, Liu J, Zhang H, Zhou L, Lin Q, Yu J, Zhang K, Liu J, Gao X (2018). Single-cell transcriptomics reveals gene signatures and alterations associated with aging in distinct neural stem/progenitor cell subpopulations. Protein Cell.

[CR85] Simon M, Van Meter M, Ablaeva J, Ke Z, Gonzalez RS, Taguchi T, De Cecco M, Leonova KI, Kogan V, Helfand SL (2019). LINE1 derepression in aged wild-type and SIRT6-deficient mice drives inflammation. Cell Metab.

[CR86] Stahl PL, Salmen F, Vickovic S, Lundmark A, Navarro JF, Magnusson J, Giacomello S, Asp M, Westholm JO, Huss M (2016). Visualization and analysis of gene expression in tissue sections by spatial transcriptomics. Science.

[CR87] Su H, Na N, Zhang X, Zhao Y (2017). The biological function and significance of CD74 in immune diseases. Inflamm Res.

[CR88] Subramanian A, Tamayo P, Mootha VK, Mukherjee S, Ebert BL, Gillette MA, Paulovich A, Pomeroy SL, Golub TR, Lander ES (2005). Gene set enrichment analysis: a knowledge-based approach for interpreting genome-wide expression profiles. Proc Natl Acad Sci.

[CR89] Sweeney MD, Sagare AP, Zlokovic BV (2018). Blood-brain barrier breakdown in Alzheimer disease and other neurodegenerative disorders. Nat Rev Neurol.

[CR90] Tanaka T, Biancotto A, Moaddel R, Moore AZ, Gonzalez-Freire M, Aon MA, Candia J, Zhang P, Cheung F, Fantoni G (2018). Plasma proteomic signature of age in healthy humans. Aging Cell.

[CR91] Tiensuu H, Haapalainen AM, Karjalainen MK, Pasanen A, Huusko JM, Marttila R, Ojaniemi M, Muglia LJ, Hallman M, Ramet M (2019). Risk of spontaneous preterm birth and fetal growth associates with fetal SLIT2. PLoS Genet.

[CR92] Tilstra JS, Clauson CL, Niedernhofer LJ, Robbins PD (2011). NF-kappaB in aging and disease. Aging Dis.

[CR93] Trapnell C, Cacchiarelli D, Grimsby J, Pokharel P, Li S, Morse M, Lennon NJ, Livak KJ, Mikkelsen TS, Rinn JL (2014). The dynamics and regulators of cell fate decisions are revealed by pseudotemporal ordering of single cells. Nat Biotechnol.

[CR94] Ulland TK, Colonna M (2018). TREM2—a key player in microglial biology and Alzheimer disease. Nat Rev Neurol.

[CR95] Vanlandewijck M, He L, Mae MA, Andrae J, Ando K, Del Gaudio F, Nahar K, Lebouvier T, Lavina B, Gouveia L (2018). A molecular atlas of cell types and zonation in the brain vasculature. Nature.

[CR96] Végh MJ, Rausell A, Loos M, Heldring CM, Jurkowski W, van Nierop P, Paliukhovich I, Li KW, del Sol A, Smit AB (2014). Hippocampal extracellular matrix levels and stochasticity in synaptic protein expression increase with age and are associated with age-dependent cognitive decline. Mol Cell Proteom.

[CR97] Volkman HE, Stetson DB (2014). The enemy within: endogenous retroelements and autoimmune disease. Nat Immunol.

[CR98] Wang L, Song G, Zhang X, Feng T, Pan J, Chen W, Yang M, Bai X, Pang Y, Yu J (2017). PADI2-mediated citrullination promotes prostate cancer progression. Cancer Res.

[CR99] Wang S, Zheng Y, Li J, Yu Y, Zhang W, Song M, Liu Z, Min Z, Hu H, Jing Y (2020). Single-cell transcriptomic atlas of primate ovarian aging. Cell.

[CR100] Wang S, Zheng Y, Li Q, He X, Ren R, Zhang W, Song M, Hu H, Liu F, Sun G (2020). Deciphering primate retinal aging at single-cell resolution. Protein Cell.

[CR101] Wegiel J, Frackowiak J, Mazur-Kolecka B, Schanen NC, Cook EH, Sigman M, Brown WT, Kuchna I, Wegiel J, Nowicki K (2012). Abnormal intracellular accumulation and extracellular Abeta deposition in idiopathic and Dup15q11.2-q13 autism spectrum disorders. PLoS One.

[CR102] Woo MS, Ufer F, Rothammer N, Di Liberto G, Binkle L, Haferkamp U, Sonner JK, Engler JB, Hornig S, Bauer S (2021). Neuronal metabotropic glutamate receptor 8 protects against neurodegeneration in CNS inflammation. J Exp Med.

[CR103] Wyss-Coray T (2016). Ageing, neurodegeneration and brain rejuvenation. Nature.

[CR104] Yang X, Goh A, Chen SH, Qiu A (2013). Evolution of hippocampal shapes across the human lifespan. Hum Brain Mapp.

[CR105] Yang AC, Stevens MY, Chen MB, Lee DP, Stahli D, Gate D, Contrepois K, Chen W, Iram T, Zhang L (2020). Physiological blood-brain transport is impaired with age by a shift in transcytosis. Nature.

[CR106] Young MD, Behjati S (2020). SoupX removes ambient RNA contamination from droplet-based single-cell RNA sequencing data. GigaScience.

[CR107] Yu HC, Tung CH, Huang KY, Huang HB, Lu MC (2020). The essential role of peptidylarginine deiminases 2 for cytokines secretion, apoptosis, and cell adhesion in macrophage. Int J Mol Sci.

[CR108] Yuan J, Amin P, Ofengeim D (2019). Necroptosis and RIPK1-mediated neuroinflammation in CNS diseases. Nat Rev Neurosci.

[CR109] Zenker M, Bunt J, Schanze I, Schanze D, Piper M, Priolo M, Gerkes EH, Gronostajski RM, Richards LJ, Vogt J (2019). Variants in nuclear factor I genes influence growth and development. Am J Med Genet C Semin Med Genet.

[CR110] Zhang W, Li J, Suzuki K, Qu J, Wang P, Zhou J, Liu X, Ren R, Xu X, Ocampo A (2015). Aging stem cells. A Werner syndrome stem cell model unveils heterochromatin alterations as a driver of human aging. Science.

[CR111] Zhang W, Wan H, Feng G, Qu J, Wang J, Jing Y, Ren R, Liu Z, Zhang L, Chen Z (2018). SIRT6 deficiency results in developmental retardation in cynomolgus monkeys. Nature.

[CR112] Zhang K, Wang Y, Fan T, Zeng C, Sun ZS (2020). The p21-activated kinases in neural cytoskeletal remodeling and related neurological disorders. Protein Cell.

[CR113] Zhang W, Qu J, Liu GH, Belmonte JCI (2020). The ageing epigenome and its rejuvenation. Nat Rev Mol Cell Biol.

[CR114] Zhang W, Zhang S, Yan P, Ren J, Song M, Li J, Lei J, Pan H, Wang S, Ma X (2020). A single-cell transcriptomic landscape of primate arterial aging. Nat Commun.

[CR115] Zhong S, Ding W, Sun L, Lu Y, Dong H, Fan X, Liu Z, Chen R, Zhang S, Ma Q (2020). Decoding the development of the human hippocampus. Nature.

[CR116] Zhou Y, Zhou B, Pache L, Chang M, Khodabakhshi AH, Tanaseichuk O, Benner C, Chanda SK (2019). Metascape provides a biologist-oriented resource for the analysis of systems-level datasets. Nat Commun.

