# Supplementary material for: Single-nucleus transcriptomic landscape of primate hippocampal aging
Source: Protein Cell. 2021 May 30;12(9):695–716. doi: 10.1007/s13238-021-00852-9 (PMC8403220; doi:10.1007/s13238-021-00852-9)
Supplement: Supplementary file 1 — Supplementary material 1 (PDF 19704 kb) [file 13238_2021_852_MOESM1_ESM.pdf]

Supplementary Figure Legends

Figure S1. Establishment of the single-nucleus transcriptomic atlas of NHP hippocampal aging.

(A) Sample information of monkeys used in this study.

(B) Staining of cell nuclei by Hoechst 33342 in DG, CA1 and CA3 regions of the hippocampus from young and old monkeys. Representative images are shown on the left; quantitative data for the relative cell density in indicated regions are shown as means ± SEM on the right. Cell density is quantified as fold changes in the old DG, CA1 and CA3 regions vs. in young counterparts. Scale bar, 50 μm. Young, n = 8; old, n = 8 monkeys. ns, not significant.

(C) Sequencing information of the snRNA-seq.

(D) UMAP plots showing distribution of cells from different groups of monkey hippocampus. YM, young male; YF, young female; OM, old male; OF, old female.

Figure S2. Age-associated gene expression analysis of monkey hippocampus across different cell types.

(A) Violin plots showing the expression levels of canonical marker genes of the indicated cell types in monkey hippocampus from young and old groups.

(B) Sankey plot showing the numbers of overlapped genes between top 30 cell-type-specific genes and disease-related genes and genes from Aging Atlas (https://bigd.big.ac.cn/aging). AA, Aging Atlas; LD, learning disorders; MD, memory disorders; AD, Alzheimer's Disease; PD, Parkinson's Disease.

(C) Line plots showing the numbers of aging-related DEGs of different cell types in monkey hippocampus from male, female, and both groups.

(D) Heatmap showing overlapped genes between aging-related DEGs and genes from Aging Atlas.

(E) Network plot showing the numbers of changed cell-cell interactions between indicated cell types in monkey hippocampus. Edge color from blue to red indicates the number from low to high.

(F) Bar plots showing the number of young-specific and old-specific cell-cell interactions between the indicated cell type with other cell types in monkey hippocampus.

Figure S3. Aging-associated gene expression alterations along the trajectories of neurogenesis.

(A) Pseudotime analysis of neuronal lineage cells in monkey hippocampus. The arrows indicate the directions of differentiation trajectories. The color from dark blue to light blue indicates the pseudotime from the beginning to the end state.

(B) Pseudotime analysis showing the expression levels of indicated genes along the trajectory of neurogenesis in monkey hippocampus.

(C) Box plot showing the proportions of TAPCs in indicated cell cycle phases in monkey hippocampus from young and old groups.

(D) Heatmap showing the target genes (column) related to learning or memory with their indicated upstream regulators (row). The genes in red represent the upregulated regulators, the blue, in turn represent the downregulated ones.

Figure S4. Cellular and molecular changes of neurogenic niche during monkey hippocampal aging.

(A) Pseudotime analysis of OPC and OL in the monkey hippocampus. The arrows indicate the directions of differentiation trajectories. The color from dark blue to light blue indicates the pseudotime from the beginning to the end state.

(B) Pseudotime analysis showing the expression levels of indicated genes along the trajectory from OPC to OL of the monkey hippocampus. The points are colored by cell type (left) and cell state (right).

(C) Box plots (bottom) showing the proportion of different subtypes of OL in the hippocampus from young and old groups.

(D) Violin plots showing SASP gene set scores in different cell types of monkey hippocampus from young and old groups.

(E) Immunohistochemical staining of TNF-α in the CA3 region of hippocampus from young and old monkeys. Representative images are shown on the left; signal intensity of TNFα is quantified as fold changes in the old CA3 regions vs. in young counterparts, shown as means ± SEM on the right. Scale bars, 20 μm and 10 μm (zoomed-in images). Young, n = 8; old, n = 8 monkeys. ns, not significant.

Supplementary Tables

Table S1. Marker genes and their upstream transcription factors across different cell types of monkey hippocampus.

Table S2. Differentially expressed genes across different cell types in monkey hippocampus between young and old groups.

Table S3. Young-specific and old-specific cell-cell interaction pairs between cell types in monkey hippocampus.

Table S4. Top 500 differentially expressed genes along the trajectories of the neurogenic lineage and oligodendrocyte lineage.

Table S5. Transcription factors regulating the aging-related differentially expressed gene of NSC and TAPC in monkey hippocampus.

Table S6. Gene lists used in this study.

Figure S1

A

No.	Species	Sex	Animal Symbol	Age (Year)
1	*Macaca fascicularis*	Male	YM1	~5
2	*Macaca fascicularis*	Male	YM2	~5
3	*Macaca fascicularis*	Male	YM3	~6
4	*Macaca fascicularis*	Male	YM4	~6
5	*Macaca fascicularis*	Female	YF1	~5
6	*Macaca fascicularis*	Female	YF2	~5
7	*Macaca fascicularis*	Female	YF3	~5
8	*Macaca fascicularis*	Female	YF4	~4
9	*Macaca fascicularis*	Male	OM1	~18
10	*Macaca fascicularis*	Male	OM2	~19
11	*Macaca fascicularis*	Male	OM3	~20
12	*Macaca fascicularis*	Male	OM4	~21
13	*Macaca fascicularis*	Female	OF1	~18
14	*Macaca fascicularis*	Female	OF2	~19
15	*Macaca fascicularis*	Female	OF3	~19
16	*Macaca fascicularis*	Female	OF4	~20

B

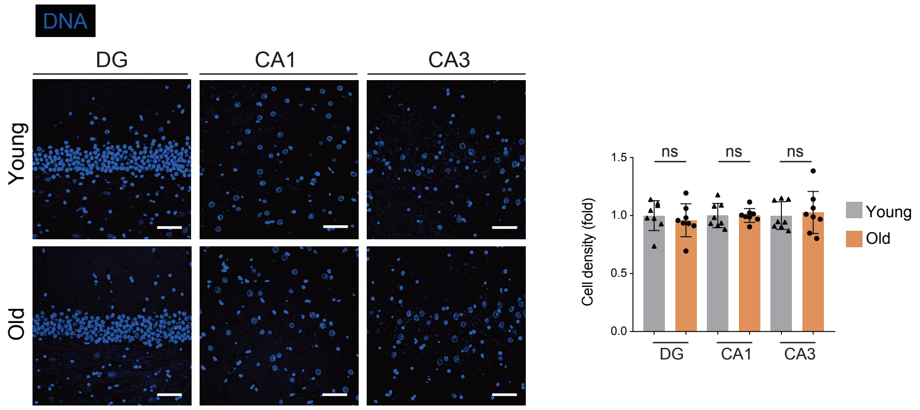

C

Animal Symbol	Sequencing saturation (%)	Mapping rate to transcriptome (%)	Doublet rate (%)
YM1	70.0	35.1	7.0
YM2	87.3	30.1	1.7
YM3	73.2	33.8	3.9
YM4	73.1	40.3	6.7
YF2	70.6	42.5	4.9
YF3	72.8	43.3	4.1
YF4	87.6	40.4	4.7
OM1	68.7	35.6	4.8
OM2	85.0	39.8	3.2
OM3	73.8	31.0	5.8
OM4	78.1	48.3	7.0
OF1	71.2	39.0	7.1
OF2	71.5	42.3	5.5
OF3	66.5	31.4	6.9
OF4	71.8	36.9	4.0

D

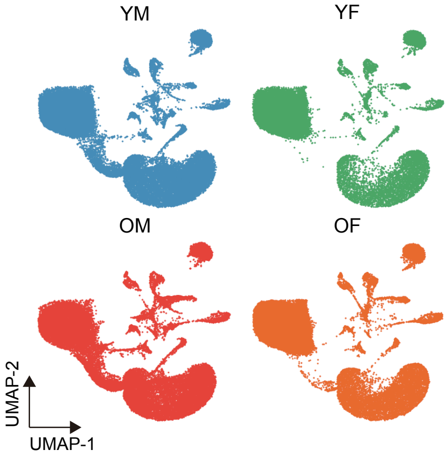

Figure S2

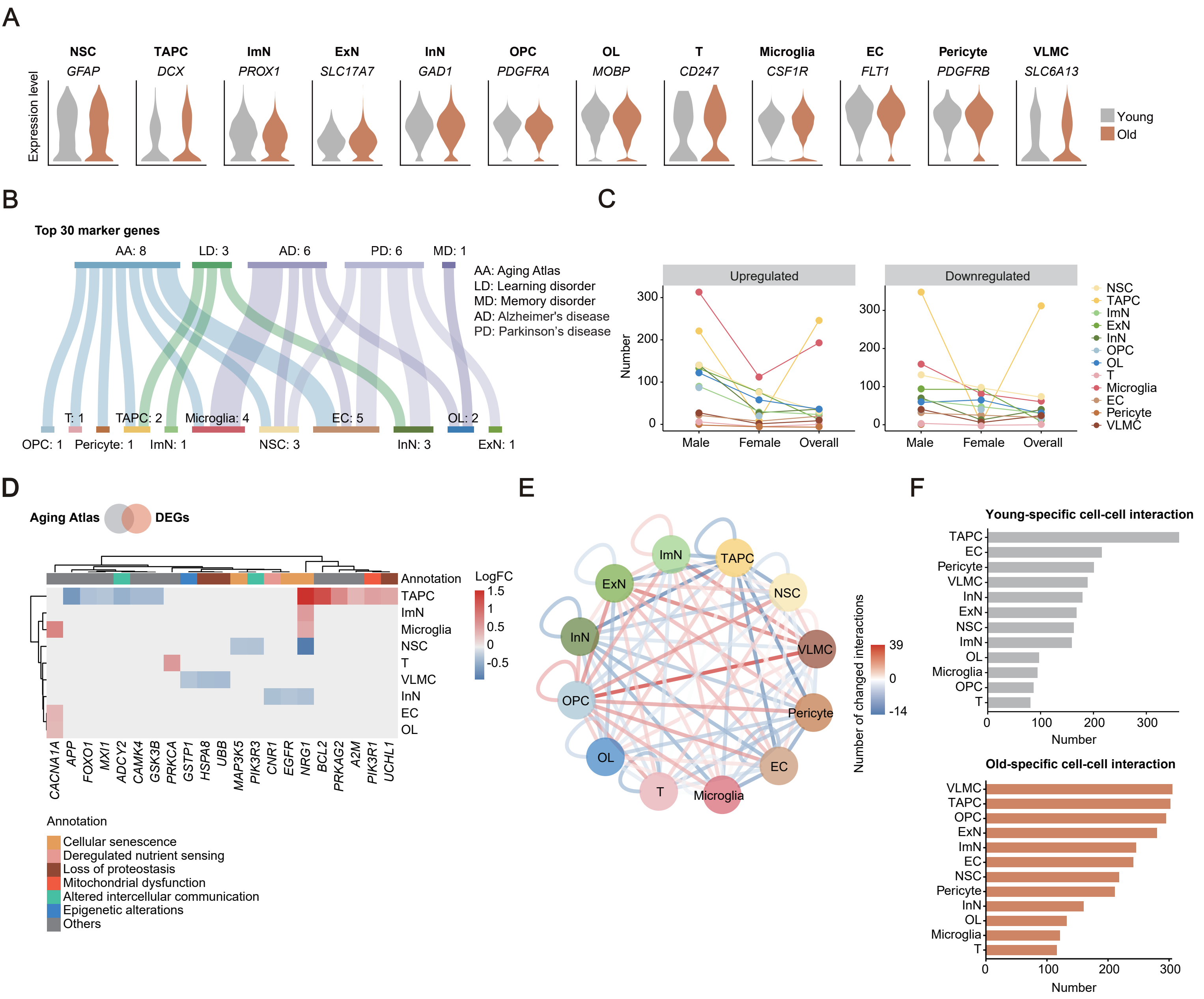
A
NSC GFAP
TAPC DCX
ImN PROX1
ExN SLC17A7
InN GAD1
OPC PDGFRA
OL MOBP
T CD247
Microglia CSF1R
EC FLT1
Pericyte PDGFRB
VLMC SLC6A13
Expression level
Young
Old
B
Top 30 marker genes
AA: 8
LD: 3
AD: 6
PD: 6
MD: 1
AA: Aging Atlas
LD: Learning disorder
MD: Memory disorder
AD: Alzheimer's disease
PD: Parkinson's disease
OPC: 1
T: 1
Pericyte: 1
TAPC: 2
ImN: 1
Microglia: 4
NSC: 3
EC: 5
InN: 3
OL: 2
ExN: 1
C
Upregulated
Downregulated
Number
Male
Female
Overall
NSC
TAPC
ImN
ExN
InN
OPC
OL
T
Microglia
EC
Pericyte
VLMC
D
Aging Atlas
DEGs
Annotation
LogFC
CACNA1A APP FOXO1 MXI1 ADCY2 CAMK4 GSK3B PRKCA GSTP1 HSPA8 UBB MAP3K5 PIK3R3 CNR1 EGFR NRG1 BCL2 PRKAG2 A2M PIK3R1 UCHL1
TAPC
ImN
Microglia
NSC
T
VLMC
InN
EC
OL
Annotation
Cellular senescence
Deregulated nutrient sensing
Loss of proteostasis
Mitochondrial dysfunction
Altered intercellular communication
Epigenetic alterations
Others
E
ImN
TAPC
ExN
NSC
InN
VLMC
OPC
Pericyte
OL
EC
T
Microglia
Number of changed interactions
39
0
-14
F
Young-specific cell-cell interaction
TAPC
EC
Pericyte
VLMC
InN
ExN
NSC
ImN
OL
Microglia
OPC
T
Number
Old-specific cell-cell interaction
VLMC
TAPC
OPC
ExN
ImN
EC
NSC
Pericyte
InN
OL
Microglia
T
Number

Figure S3

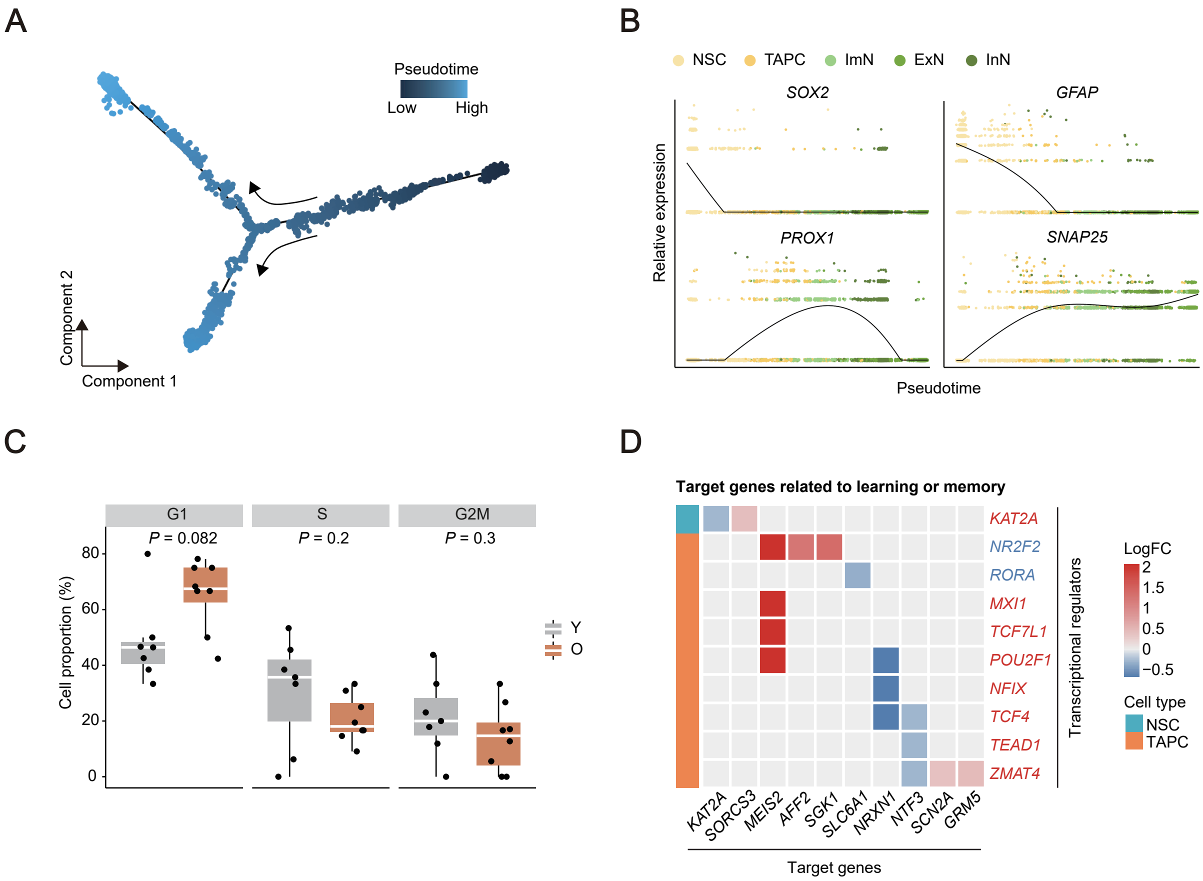
A
B
C
D
Pseudotime
Low
High
Component 2
Component 1
NSC
TAPC
ImN
ExN
InN
SOX2
GFAP
PROX1
SNAP25
Relative expression
Pseudotime
G1
S
G2M
P = 0.082
P = 0.2
P = 0.3
Cell proportion (%)
Y
O
Target genes related to learning or memory
KAT2A
NR2F2
RORA
MXI1
TCF7L1
POU2F1
NFIX
TCF4
TEAD1
ZMAT4
Transcriptional regulators
LogFC
Cell type
NSC
TAPC
KAT2A
SORCS3
MEIS2
AFF2
SGK1
SLC6A1
NRXN1
NTF3
SCN2A
GRM5
Target genes

Figure S4

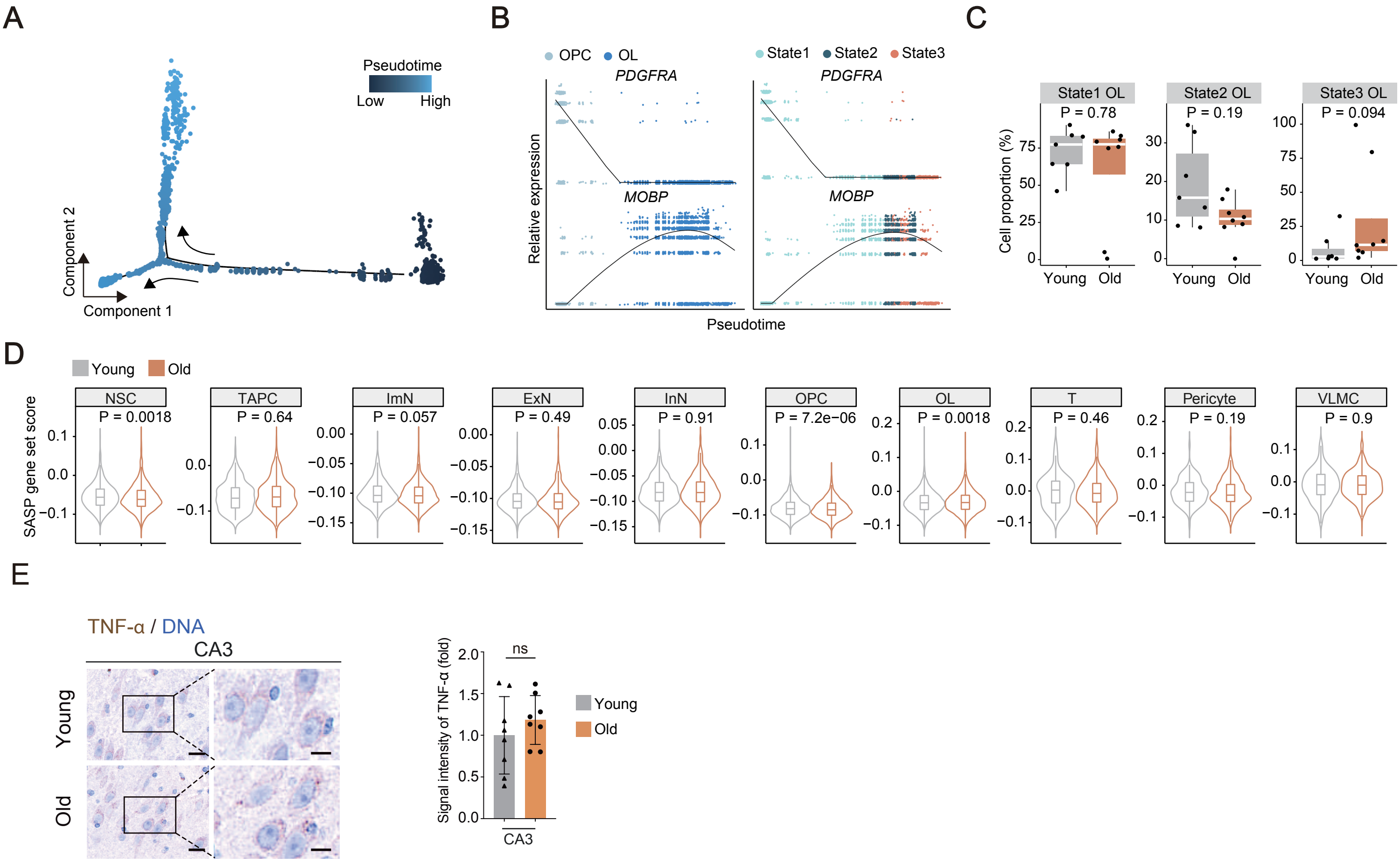
A
Pseudotime
Low
High
Component 2
Component 1
B
OPC
OL
State1
State2
State3
PDGFRA
MOBP
Relative expression
Pseudotime
C
State1 OL
State2 OL
State3 OL
P = 0.78
P = 0.19
P = 0.094
Cell proportion (%)
Young
Old
D
Young
Old
SASP gene set score
NSC
P = 0.0018
TAPC
P = 0.64
ImN
P = 0.057
ExN
P = 0.49
InN
P = 0.91
OPC
P = 7.2e−06
OL
P = 0.0018
T
P = 0.46
Pericyte
P = 0.19
VLMC
P = 0.9
E
TNF-α / DNA
CA3
Young
Old
Signal intensity of TNF-α (fold)
ns
CA3
Young
Old
